# Time-stratified prognostic factors for poor renal outcomes in childhood-onset lupus nephritis: a systematic review and meta-analysis

**DOI:** 10.3389/fimmu.2026.1939149

**Published:** 2026-09-25

**Authors:** Yuanhui Hu, Hailong Li, Weiqi Deng, Sha Diao, Linan Zeng, Liang Huang, Jingjing Chen, Yuhong Tao, Lingli Zhang

**Affiliations:** 1 West China School of Pharmacy, Sichuan University, Chengdu, China; 2 Department of Pharmacy/Evidence-Based Pharmacy Center, West China Second University Hospital, Sichuan University, Chengdu, China; 3 Children’s Medicine Key Laboratory of Sichuan Province, Chengdu, China; 4 NMPA Key Laboratory for Technical Research on Drug Products In Vitro and In Vivo Correlation, Chengdu, China; 5 Key Laboratory of Birth Defects and Related Diseases of Women and Children, Sichuan University, Ministry of Education, Chengdu, China; 6 West China Lecheng Hospital of Sichuan University, Chengdu, China; 7 West China Biomedical Big Data Center, West China Hospital, Sichuan University, Qionghai, China; 8 Department of Pediatrics, West China Second University Hospital, Sichuan University, Chengdu, China; 9 Chinese Evidence-based Medicine Center, West China Hospital, Sichuan University, Chengdu, China

**Keywords:** childhood-onset lupus nephritis, meta-analysis, poor renal outcomes, prognostic factors, risk stratification

## Abstract

**Objectives:**

To identify prognostic factors independently associated with poor renal outcomes in childhood-onset lupus nephritis (cLN) based on multivariable analyses.

**Methods:**

Seven databases were searched up until October 2025. Adjusted odds ratio (OR), hazard ratios (HR), and pooled proportions of renal outcomes were estimated using random-effects models. Outcome-specific analyses were performed when sufficient data were available, and sensitivity analyses excluding studies at high risk of bias were conducted to assess the robustness of findings. Methodological quality was evaluated using quality in prognostic studies tool. Certainty of evidence was assessed using Grading of Recommendations, Assessment, Development and Evaluation.

**Results:**

47 studies were included, with 23 assessed as having a high risk of bias. The included studies explored prognostic factors across follow-up periods of 1 year (n=11), 1–2 years (n=9), and >2 years (n=27). Within 1 year, 27 (OR) prognostic factors were identified. Within 1–2 years, 25 (OR) and 6 (HR) prognostic factors were identified respectively. Beyond 2 years, 26 (OR) and 55 (HR) were identified respectively. Frequently evaluated factors associated with poor renal outcomes included age, dialysis, eGFR <60, hypertension, kidney flare, male sex, and renal non-remission within 6 or 12 months. Nephrotic-range proteinuria and increased serum creatinine were associated with poor renal outcomes in the primary analysis but showed limited robustness after excluding studies at high risk of bias. Higher eGFR and hemoglobin levels showed inverse associations with poor renal outcomes. Outcome-specific analyses suggested that male sex and increased serum creatinine were mainly associated with kidney-specific outcomes comprising CKD and/or ESRD, whereas renal non-remission at 6 and 12 months was associated with composite outcomes. Except for renal non-remission (1 year: 23.00%; >2 years: 22.43%), the reported cumulative proportions of other outcomes were higher in studies with longer follow-up durations (CKD: 4.19% to 11.46%; ESRD: 3.10% to 8.58%; mortality: 2.69% to 8.71%).

**Conclusion:**

Identified prognostic factors provide a reference for stratified management of cLN. Differences in prognostic associations across studies categorized by follow-up duration and higher cumulative proportions of adverse outcomes in studies with longer follow-up highlight the need for longitudinal, dynamic risk assessment strategies.

**Systematic review registration:**

https://www.crd.york.ac.uk/PROSPERO/view/CRD420251169446, identifier CRD420251169446.

## Graphical Abstract

**Figure:**
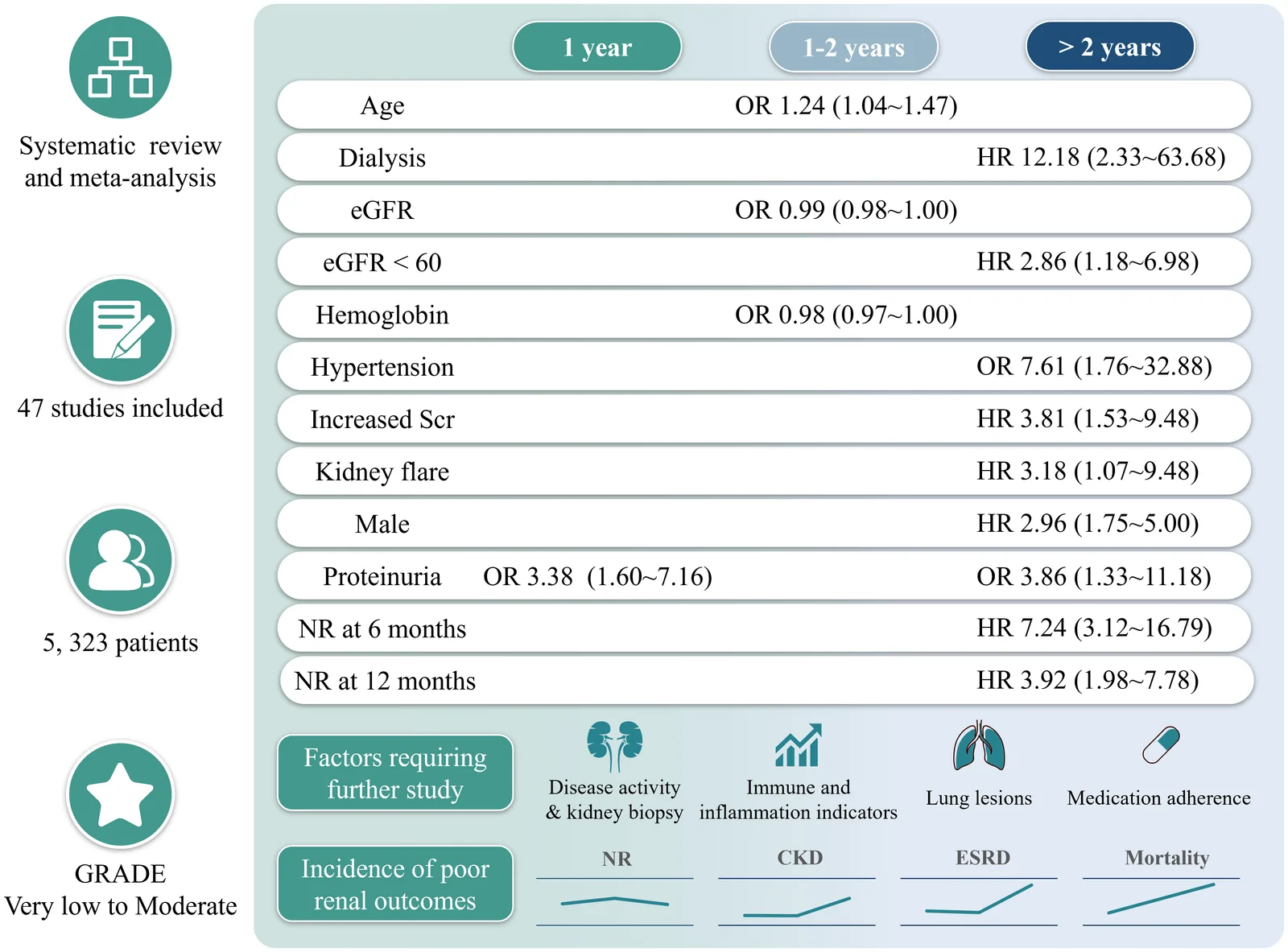

## 1 Introduction

Lupus nephritis (LN) is the most prevalent and severe complication of systemic lupus erythematosus (SLE) in children, affecting approximately 50–60% of pediatric SLE patients (1, 2). Compared with children with SLE without renal involvement, those with childhood-onset lupus nephritis (cLN) face a substantially higher risk of poor treatment response, chronic kidney disease (CKD), and end-stage renal disease (ESRD), with mortality risk 22 times that of the healthy pediatric population (3–5). Although advances in immunosuppressive and biologic therapies have expanded treatment options, complete renal response rates within 1–2 years generally remain below 50% in major clinical trials (2, 6–8). Moreover, during long-term follow-up, up to 9% of patients die and 14% progress to ESRD (9, 10), and this proportion has remained stable over the past decade (11), resulting in significant impairment of quality of life and substantial socioeconomic burden (12, 13). Therefore, the management of cLN should address both the achievement of an early renal response and the prevention of cumulative, irreversible kidney damage over time.

Early identification of high-risk individuals is critical for individualized treatment and improved prognosis (14). However, prognostic associations reported at different follow-up periods may differ. For example, in an inception cohort of patients with proliferative LN, lower baseline proteinuria predicted complete renal response at 3, 6, and 9 months, but was not associated with long-term renal survival. In contrast, baseline interstitial fibrosis and tubular atrophy did not predict early complete renal response, but was associated with the development of CKD or ESRD during long-term follow-up (15). Another long-term study similarly showed that the prognostic value of laboratory and disease activity measures changed over time (16). Thus, factors associated with short-term outcomes may differ from those reported for long-term kidney outcomes. Combining evidence from studies with markedly different follow-up durations may therefore obscure clinically important differences in prognostic value, supporting separate evaluation according to follow-up duration.

Although numerous studies have explored prognostic factors for poor renal outcomes in cLN, their findings remain inconsistent, partly because of small sample sizes, heterogeneous outcome definitions and follow-up durations, and differences in covariate adjustment. A 2021 meta-analysis examined prognostic factors in LN (17); however, it did not specifically focus on pediatric populations, assess the certainty of evidence, or use dedicated tools for assessing risk of bias in prognostic studies. Importantly, it did not distinguish between short-term and long-term prognostic factors. These limitations restrict the application of existing evidence to time-specific risk assessment and clinical decision-making in children with LN.

Therefore, this systematic review and meta-analysis aimed to identify and synthesize prognostic factors independently associated with poor renal outcomes after multivariable adjustment in cLN across different follow-up periods and to evaluate the certainty of the evidence. We also estimated pooled proportions of major poor renal outcomes across predefined follow-up duration categories.

## 2 Method

This study was prospectively registered with PROSPERO (CRD420251169446) and reported following the Preferred Reporting Items for Systematic Reviews and Meta-Analyses (PRISMA) guidelines (18). The completed PRISMA checklist is provided in **Supplementary Table S1**.

### 2.1 Searching strategy

Two reviewers (Y.H. Hu and S. Diao) independently searched seven databases—China National Knowledge Infrastructure, Chinese Biomedical Literature Database, Wanfang Database, VIP, PubMed, Embase, and Scopus—from inception to October 10, 2025, without language restrictions. Search terms included “lupus nephritis,” “child,” “prognos*,” and “risk*”; the complete search strategies are provided in **Supplementary Table S2**.

### 2.2 Study selection

Two reviewers (Y.H. Hu and S. Diao) independently screened records using Covidence, with disagreements resolved by a third reviewer (H.L. Li). Studies were eligible if they included patients with SLE diagnosed according to established classification criteria, including the American College of Rheumatology (ACR) criteria, the Systemic Lupus International Collaborating Clinics (SLICC) criteria, or the European League Against Rheumatism/American College of Rheumatology (EULAR/ACR) criteria, and LN diagnosed based on clinical criteria and/or kidney biopsy findings at ≤18 years of age; evaluated clinical factors potentially associated with poor renal outcomes, defined as renal non-remission, CKD, ESRD, or LN-related mortality, either individually or as composite outcomes; and had a minimum follow-up of 6 months. Studies were excluded if they were non-primary publications, reported insufficient data for meta-analysis, included only univariable analyses, or were duplicate publications.

### 2.3 Data extraction and quality assessment

Two reviewers (Y.H. Hu and S. Diao) independently extracted first author, country/region, publication year, study design, sample size, main kidney biopsy class, age at LN diagnosis, follow-up duration, number of events for each poor renal outcomes, statistical method, and main findings using a pre-piloted form. Disagreements were resolved by a third reviewer (H.L. Li). When multiple multivariable models were reported for the same cohort and outcome, only the estimate from the most fully adjusted model was selected. When the same cohort reported different renal outcomes derived from the same patient population, these estimates were combined using the reported effect estimates and variances on the log scale to generate a single cohort-level effect estimate before meta-analysis. When both overall and subgroup analyses were available, only the overall-population estimate was extracted.

Risk of bias was assessed using the Quality in Prognostic Studies (QUIPS) tool (19). Studies were classified as low risk if all domains were rated as low risk or only one domain was rated as moderate risk; as high risk if at least one domain was rated as high risk or three or more domains were rated as moderate risk; and as moderate risk in all other cases.

### 2.4 Data analysis

Study characteristics were summarized descriptively. Adjusted effect estimates were pooled separately as odds ratio (OR) or hazard ratio (HR), with 95% confidence interval (CI), using random-effects models. Unless otherwise specified, continuous prognostic factors were presented per one-unit increase in the corresponding measurement scale. Consistent with the 2024 KDIGO guideline for LN management, which recommends assessing initial treatment response at 6–12 months and allows extension to 18–24 months in patients with ongoing improvement (14), follow-up was categorized as ≤1 year, 1–2 years, and >2 years. These categories represent the initial response phase, the period of delayed or continued response, and long-term outcomes beyond the expected response-assessment window, respectively. Follow-up duration will be based on the median or average follow-up time. If no information is available, the shortest follow-up time specified in the included studies will be used. Given the clinical heterogeneity of poor renal outcomes, outcomes were further grouped into four categories: renal non-remission; kidney-specific outcomes comprising CKD and/or ESRD; mortality alone; and composite outcomes. Outcome-specific meta-analysis was conducted when at least two studies reported the same prognostic factor for the same outcome category within the same follow-up period and using the same effect measure. For studies reporting relative risk, relative risk was considered approximately equivalent to OR based on the rare-outcome assumption, as these estimates were primarily derived from long-term studies evaluating relatively uncommon outcomes such as ESRD or mortality (20), and effect estimates for renal remission were inverted to represent poor renal outcomes. Only prognostic factors reported by at least two studies were described in the results. The pooled proportions of each poor renal outcome were estimated across predefined follow-up duration categories using random-effects models after logit transformation.

Heterogeneity was assessed using the I² statistic, with values of <50%, 50%–74%, and ≥75% indicating low, moderate, and high heterogeneity, respectively. Publication bias was evaluated using Egger’s test when at least 10 studies were available. All tests were two-sided, with P<0.05 considered statistically significant. Analyses were performed using SPSS 18.0, R 4.5.0, and RevMan 5.3.

### 2.5 Certainty of the evidence assessment

The certainty of evidence for frequently evaluated prognostic factors was assessed using the Grading of Recommendations Assessment, Development and Evaluation (GRADE) approach (21), considering risk of bias, inconsistency, indirectness, imprecision, and publication bias. Consistent with previous prognostic-factor meta-analyses (22, 23), frequently evaluated prognostic factors were operationally defined as those reported by at least two studies within the same follow-up period. When the same prognostic factor was evaluated using both OR and HR, the analysis with the larger sample size or lower heterogeneity was used for GRADE assessment.

### 2.6 Sensitivity analysis

Two sensitivity analyses were performed. First, when at least 10 studies were available within a follow-up period, analyses were restricted to the most commonly reported outcome. Second, studies at high risk of bias were excluded.

## 3 Results

A total of 6,406 articles were retrieved from the database. After removing duplicate articles and screening based on titles and abstracts, 415 studies were assessed in full text. Ultimately, 47 articles were included. The main reasons for exclusion were that the study population did not exclusively comprise pediatric patients (n=223) and that only univariate analyses were conducted (n=84). The literature screening process is presented in **Figure 1**.

**Figure 1:**
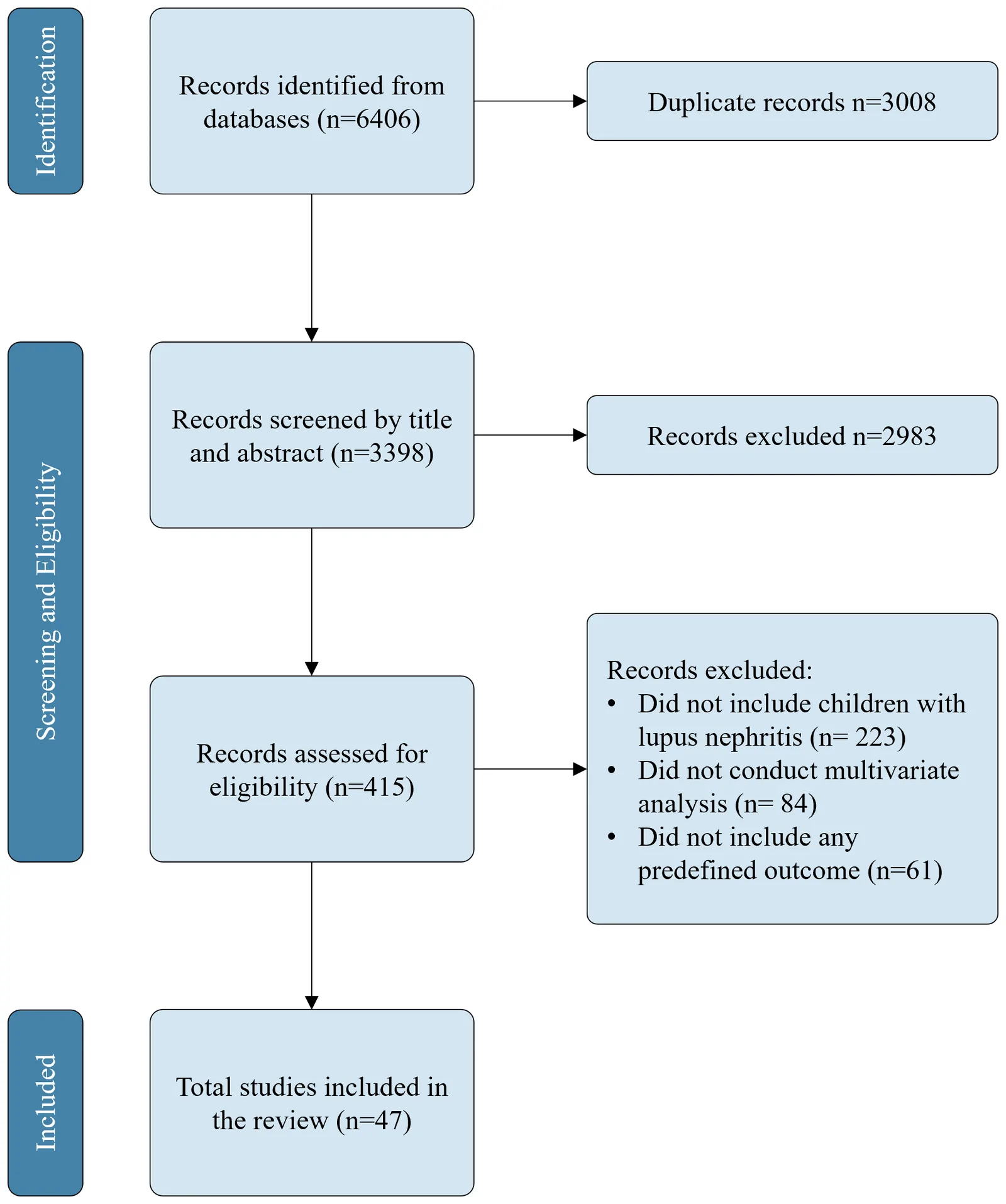
Flow diagram of study selection.

### 3.1 Study characteristics and quality assessment

A total of 47 studies (involving 5,323 patients) from 14 countries were published between 2001 and 2025, with the majority of studies conducted in China (n=26). Kidney biopsy findings were reported in most included studies, and 35 studies predominantly included children with class IV LN; five studies did not report specific biopsy classifications. Study designs included cohort studies (n=42, including 7 prospective cohort studies) and case–control studies (n=5). Among the included studies, 23 used multivariate Cox regression, 23 used multivariate logistic regression and 1 used both Cox and logistic regression. Follow-up durations ranged from 6 months to 10 years after treatment initiation. Among the 47 studies, 6 did not report the specific SLE classification criteria used; 6 used the 2012 SLICC criteria; 27 used the 1982 or 1997 ACR criteria; 2 used either the 1997 ACR criteria and/or the 2012 SLICC criteria; and 6 used the 2019 EULAR/ACR criteria. The pooled proportions of four poor renal outcomes across predefined follow-up duration categories are presented in **Figure 2**. The pooled proportion of renal non-remission remained relatively stable across follow-up duration categories (6–12 months: 23.00%; 12–24 months: 28.64%; >24 months: 22.43%). In contrast, studies with longer follow-up durations reported higher pooled proportions of other poor renal outcomes, including CKD (4.19% to 11.46%), ESRD (3.10% to 8.58%), and mortality (2.69% to 8.71%). Forest plots of the pooled proportions for all poor renal outcomes are presented in **Supplementary Figure S1**–**S4**. The baseline characteristics and detailed results of all included studies are summarized in **Supplementary Table S3**. The adjusted covariate sets, outcome measures, and their definitions for each included study are presented in **Supplementary Table S4**.

**Figure 2:**
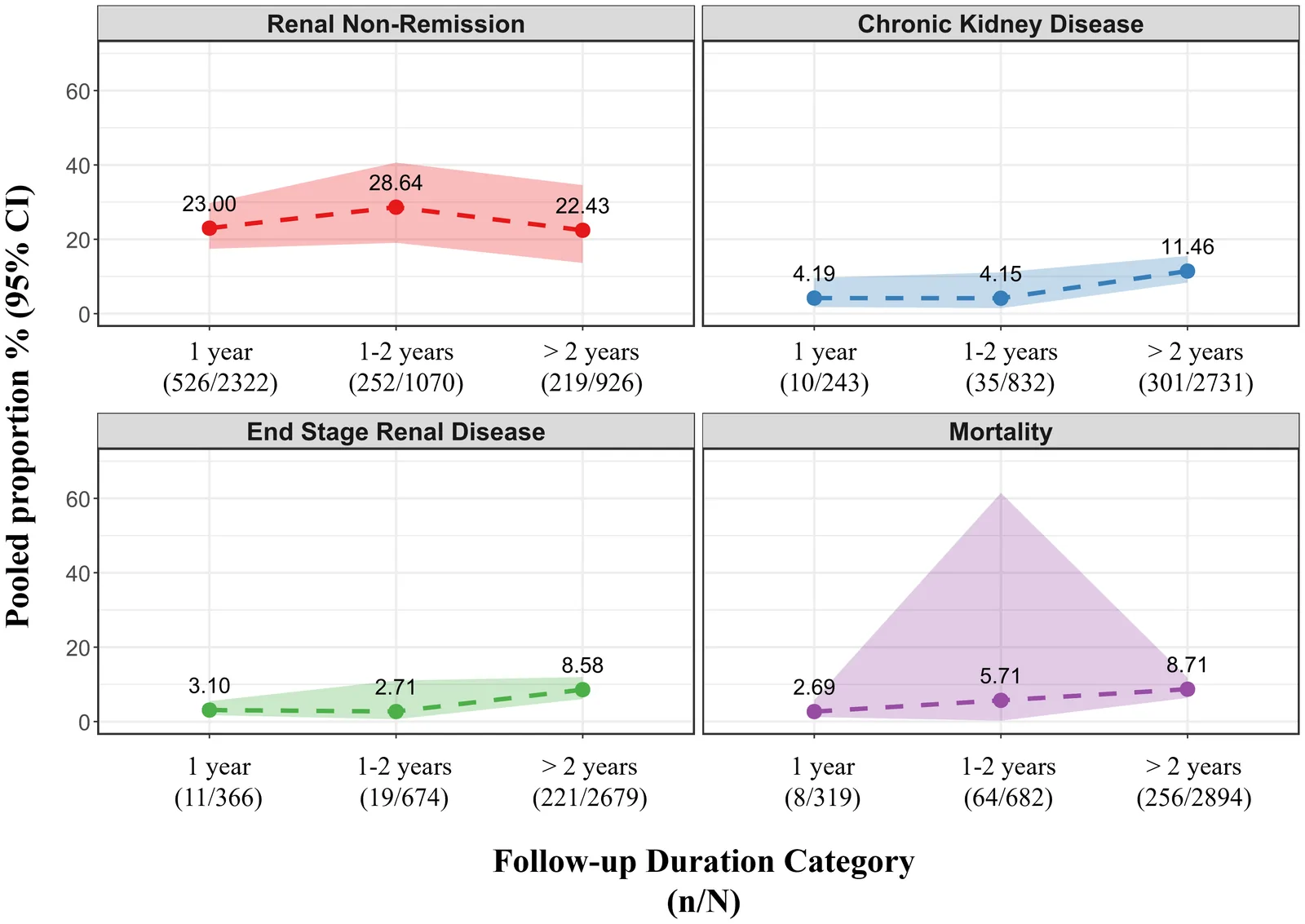
Pooled proportions of poor renal outcomes by follow-up duration categories. n/N represents the number of patients with the outcome divided by the total number of patients contributing outcome data for each outcome and follow-up category. Denominators are outcome- and follow-up category-specific and therefore do not equal the total population of 5,323 patients, as not all studies reported all outcomes. Follow-up categories represent study-level follow-up durations rather than longitudinal changes within individual patients.

Based on the QUIPS tool, 12 studies were rated as having a low risk of bias, 12 as having a moderate risk of bias, and 23 as having a high risk of bias (**Supplementary Table S5**).

### 3.2 Overall prognostic associations with poor renal outcomes

#### 3.2.1 Prognostic factors of poor renal outcomes within 1 year

Among the 47 included studies, 11 studies involving 928 patients reported prognostic factors for poor renal outcomes in cLN within one year of follow-up. After excluding one study due to data errors (**Supplementary Table S5**), all remaining studies reported effect estimates as OR, and 27 prognostic factors were identified. The most frequently evaluated factors associated with poor renal outcomes after multivariable adjustment included male and nephrotic range proteinuria (**Figure 3**). Forest plots for each prognostic factor are presented in **Supplementary Figure S5**.

**Figure 3:**
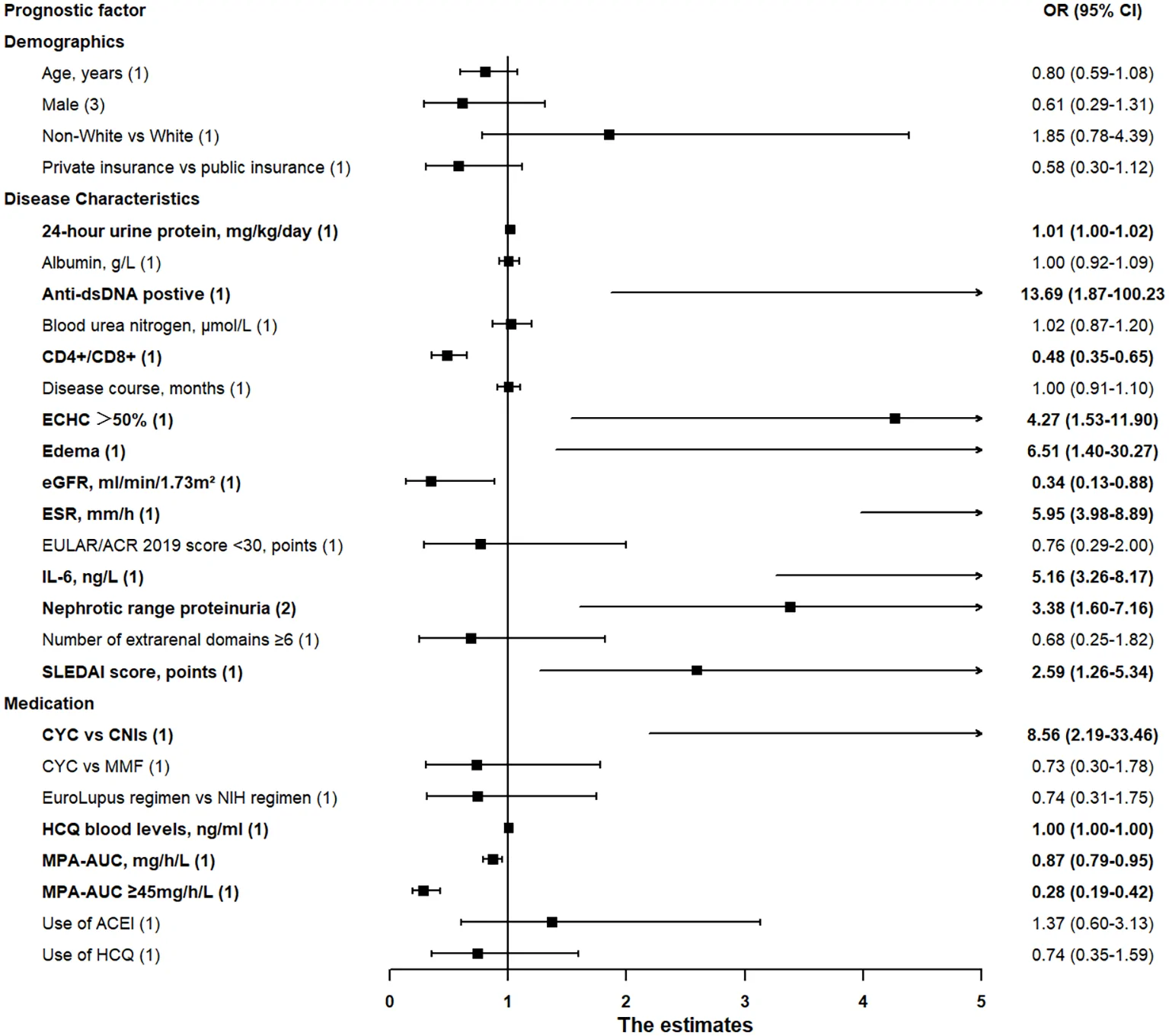
Prognostic factors of poor renal outcomes within 1 year based on data from OR (number of studies). Numbers in parentheses indicate the number of studies contributing to each estimate. Estimates based on ≥2 studies represent pooled meta-analytic estimates, whereas estimates based on a single study represent individual adjusted estimates. ACEI, angiotensin-converting enzyme inhibitor; Anti-dsDNA, anti-double-stranded DNA; CNIs, calcineurin inhibitors; CYC, cyclophosphamide; ECHC, endocapillary hypercellularity; eGFR, estimated glomerular filtration rate; ESR, erythrocyte sedimentation rate; EULAR/ACR, European league against rheumatism/American College of Rheumatology; HCQ, hydroxychloroquine; IL-6, interleukin-6; MMF, mycophenolate mofetil; MPA-AUC, mycophenolic acid-area under concentration-time curve; NIH, national institutes of health; SLEDAI, systemic lupus erythematosus disease activity index.

All 10 studies used renal remission/non-remission as the outcome. Among them, 9 defined it based on estimated glomerular filtration rate (eGFR) and proteinuria (or urine protein-to-creatinine ratio, UPCR), while 1 defined it by the need for intensified treatment.

#### 3.2.2 Prognostic factors of poor renal outcomes within 1–2 years

**Figure 4** summarizes the prognostic factors associated with poor renal outcomes in cLN during the 1–2-year follow-up period, based on 9 out of 47 studies (1,245 patients). After excluding 2 studies with data errors (**Supplementary Table S5**), remaining studies reported effect estimates as OR or HR, with 25 prognostic factors identified based on OR and 6 based on HR. The most frequently evaluated factors associated with poor renal outcomes after multivariable adjustment included age at diagnosis, biopsy class, disease course, eGFR, hemoglobin, and anti-double-stranded DNA (Anti-dsDNA) antibody. Forest plots for each prognostic factor are presented in **Supplementary Figure S6**, **S7**.

**Figure 4:**
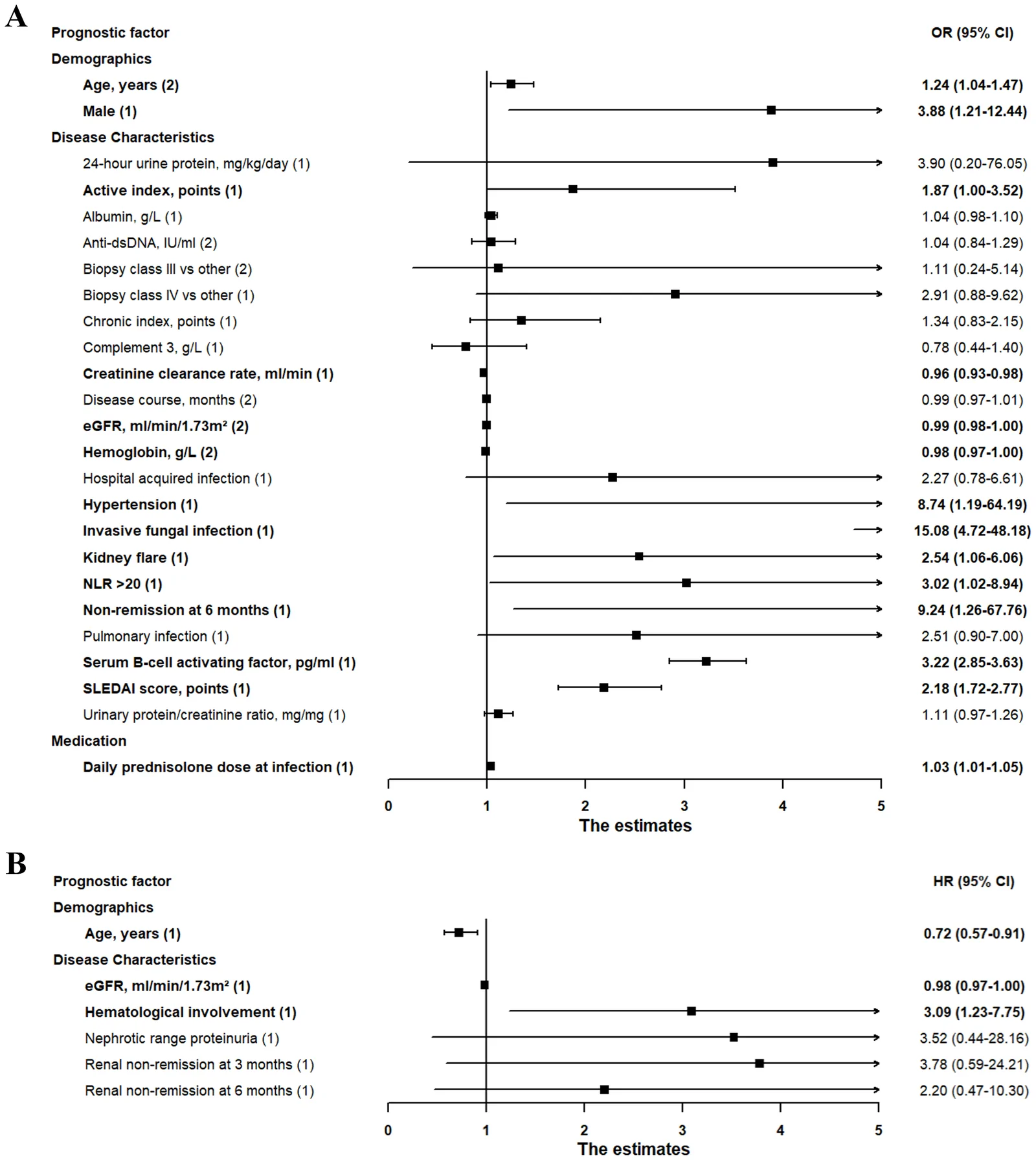
Prognostic factors of poor renal outcomes within 1–2 years based on data from OR **(A)** and HR **(B)** (number of studies). Numbers in parentheses indicate the number of studies contributing to each estimate. Estimates based on ≥2 studies represent pooled meta-analytic estimates, whereas estimates based on a single study represent individual adjusted estimates. Anti-dsDNA, anti-double-stranded DNA; eGFR, estimated glomerular filtration rate; NLR, neutrophil-to-lymphocyte ratio; SLEDAI, systemic lupus erythematosus disease activity index.

Among the 7 studies, 4 defined the outcome as either ESRD, CKD, or mortality, alone or in combination, while 3 used renal remission/non-remission as the outcome.

#### 3.2.3 Prognostic factors of poor renal outcomes for > 2 years

Among the 47 studies, 27 studies involving 3,150 patients had a follow-up period of > 2 years. After excluding 5 studies with data errors (**Supplementary Table S5**), **Figures 5** and **6** summarize 26 and 55 prognostic factors based on OR and HR, respectively. Among the studies contributing OR-based estimates, one study (24) originally reported a relative risk for ESRD, which was treated as approximately equivalent to the OR under the rare-outcome assumption before quantitative synthesis. The most frequently evaluated factors associated with poor renal outcomes after multivariable adjustment included male, biopsy class, hematological involvement, neurological involvement, hypertension, serum creatinine (Scr), eGFR <60 ml/min/1.73m², hypoproteinemia, need for dialysis at baseline, systemic lupus erythematosus disease activity index (SLEDAI) score, treatment non-compliance, kidney flare, and renal non-remission at 6 and 12 months. Forest plots for each prognostic factor are presented in **Supplementary Figure S8**, **S9**.

**Figure 5:**
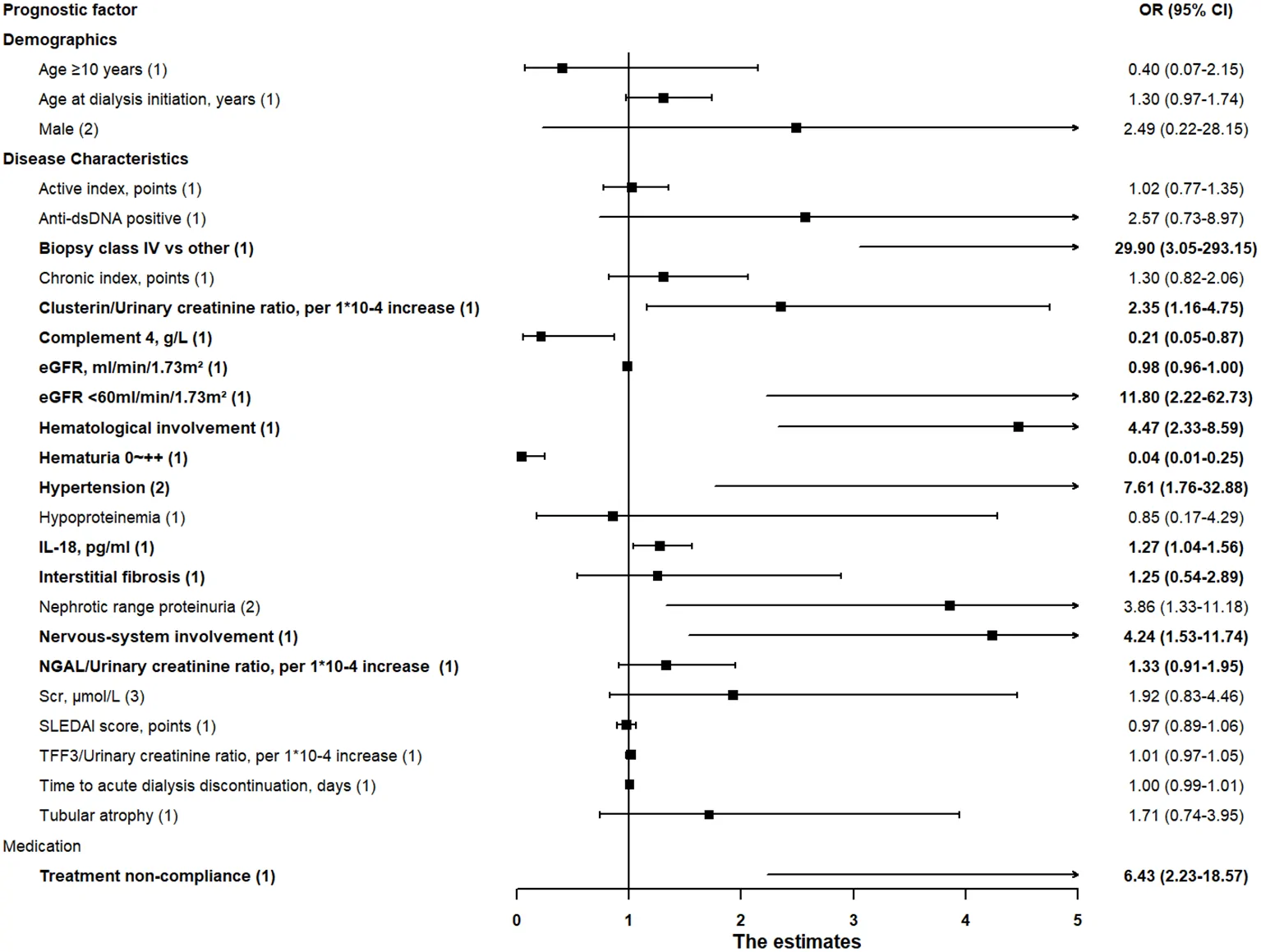
Prognostic factors of poor renal outcomes over 2 years based on data from OR (number of studies). Numbers in parentheses indicate the number of studies contributing to each estimate. Estimates based on ≥2 studies represent pooled meta-analytic estimates, whereas estimates based on a single study represent individual adjusted estimates. Anti-dsDNA, anti-double-stranded DNA; eGFR, estimated glomerular filtration rate; IL-18, interleukin-18; MPA-AUC, mycophenolic acid-area under concentration-time curve; NGAL, neutrophil gelatinase-associated lipocalin; NLR, neutrophil-to-lymphocyte ratio; Scr, serum creatinine; SLEDAI, systemic lupus erythematosus disease activity index; TFF3, trefoil factor 3.

**Figure 6:**
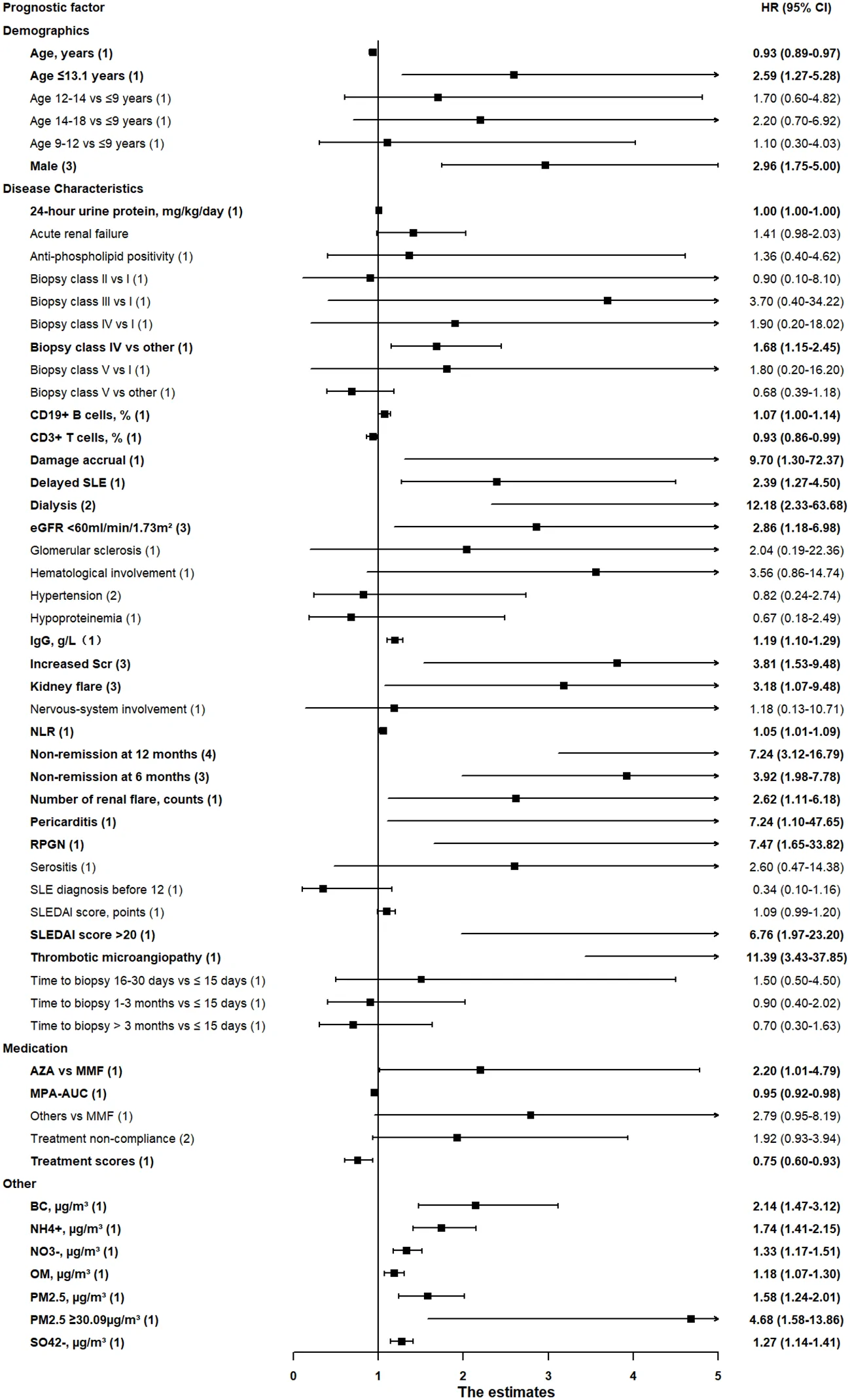
Prognostic factors of poor renal outcomes over 2 years based on data from HR (number of studies). Numbers in parentheses indicate the number of studies contributing to each estimate. Estimates based on ≥2 studies represent pooled meta-analytic estimates, whereas estimates based on a single study represent individual adjusted estimates. AZA, azathioprine; BC, black carbon; eGFR, estimated glomerular filtration rate; IgG, immunoglobulin G; MMF, mycophenolate mofetil; MPA-AUC, mycophenolic acid-area under concentration-time curve; NH4+, ammonium; NO3-, nitrate; OM, organic matter; PM2.5, particulate matter with an aerodynamic diameter ≤ 2.5µm; RPGN, rapidly progressive glomerulonephritis; Scr, serum creatinine; SLE, systemic lupus erythematosus; SLEDAI, systemic lupus erythematosus disease activity index; SO42-, sulfate.

Among the 22 studies, 18 defined the outcome as either ESRD, CKD, or mortality, alone or in combination; 2 used renal non-remission as the outcome; and 2 employed a mixed combination of these two outcome types.

Different prognostic associations were observed across follow-up categories (**Figure 7**). Prognostic indicators for short-term follow-up primarily focus on acute active injuries, such as disease activity and baseline renal function. In contrast, prognostic indicators for long-term follow-up also consider chronic cumulative injuries, including complications, immune markers, and inflammatory indicators.

**Figure 7:**
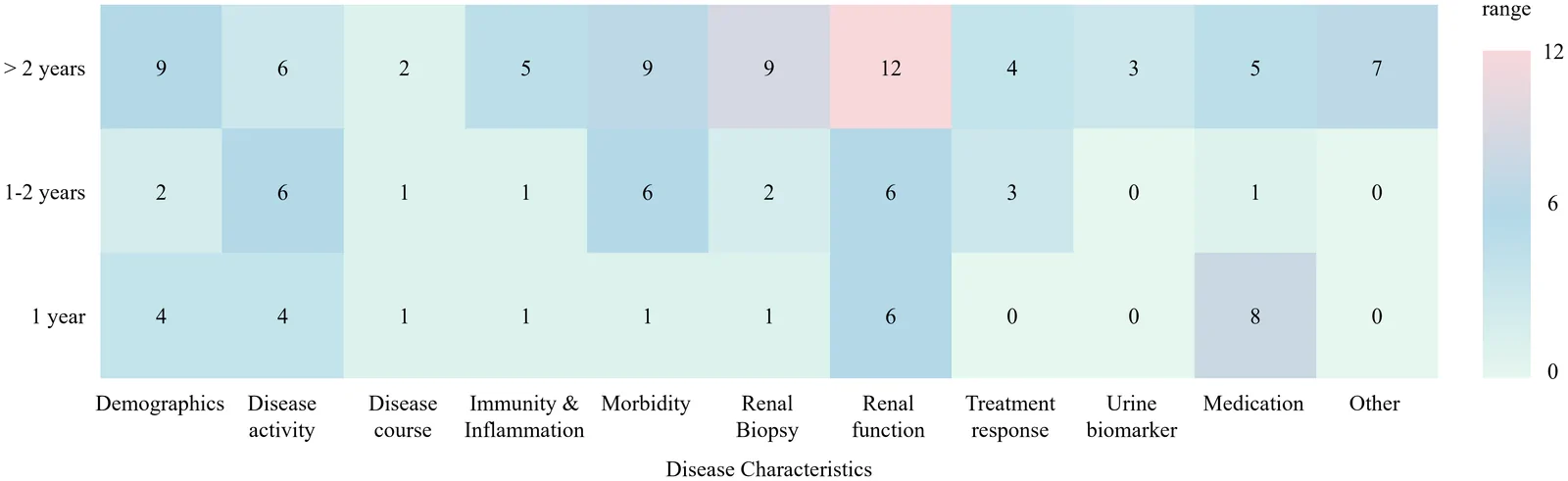
Number and category of prognostic factors identified in different follow-up periods.

##### Demographics

###### Age at diagnosis

Five studies evaluated the association between age as a continuous variable and poor renal outcomes. The pooled results of two studies suggested that age was likely associated with poor renal outcomes within 1–2 years of follow-up (OR = 1.24, 95% *CI* 1.04–1.47). However, the other two studies, one with 1–2 years of follow-up and the other with over 2 years (long-term follow-up), reported opposite results (HR = 0.72, 95% *CI* 0.57–0.91; HR = 0.93, 95% *CI* 0.89–0.97). One study found no clear evidence that age was associated with poor renal outcomes within 1 year of follow-up (OR = 0.80, 95% *CI* 0.59–1.08).

###### Male

One study found that male gender may be associated with poor renal outcomes within 1–2 years of follow-up (OR = 3.88, 95% *CI* 1.21–12.44), which was consistent with the pooled results from 3 studies with follow-up periods over 2 years (HR = 2.96, 95% *CI* 1.75–5.00). However, another 2 studies did not observe this association between gender and long-term poor renal outcomes (OR = 2.49, 95%*CI* 0.22–28.15). The pooled results of the 3 studies suggested that male gender was likely not associated with poor renal outcomes within 1 year of follow-up (OR = 0.61, 95% *CI* 0.29–1.31).

##### Disease characteristics

###### 24-hour urine protein

Two individual studies suggested that higher 24-hour urine protein levels may be associated with poor renal outcomes during a follow-up period of 1 year or over 2 years (OR = 1.01, 95% *CI* 1.00–1.02; HR = 1.00, 95% *CI* 1.00–1.00). Another study suggested that it might not be associated with poor renal outcomes occurring within 1–2 years (OR = 3.90, 95% *CI* 0.20–76.05).

###### Active index

Two studies evaluated the association between active index and poor renal outcomes. The result showed that a higher active index may be associated with poor renal outcomes within 1–2 years (OR = 1.87, 95% *CI* 1.00–3.52) but not with long-term prognosis (OR = 1.02, 95% *CI* 0.77–1.35).

###### Albumin/Hypoproteinemia at baseline

Two separate studies evaluated baseline serum albumin levels and hypoproteinemia, but no clear association with poor renal outcomes was observed.

###### Anti-dsDNA

The pooled results of two studies indicated that anti-dsDNA was unlikely to be associated with poor renal outcomes within 1–2 years of follow-up (OR = 1.04, 95% *CI* 0.84–1.29). In contrast, in the other two studies, where anti-dsDNA was treated as a binary variable, the results suggested that anti-dsDNA positivity might be associated with the occurrence of poor renal outcomes within 1 year (OR = 13.69, 95% *CI* 1.87–100.23), but was not related to long-term prognosis (OR = 2.57, 95% CI 0.73–8.97).

###### Biopsy class

The pooled results of two studies indicated that biopsy class III (compared to other types) was not clearly associated with poor renal outcomes within 1–2 years (OR = 1.11, 95% *CI* 0.24–5.14). Three studies evaluated the association between biopsy class IV (compared with other types) and poor renal outcomes. However, effect estimates were available from only two long-term follow-up studies, which suggested that biopsy class IV may be associated with poor renal outcomes (OR = 29.90, 95% CI 3.05–293.15; HR = 1.68, 95% CI 1.15–2.45).

###### Chronic index

Two studies evaluated the association between the chronic index and poor renal outcomes, but no clear association was observed.

###### Dialysis at diagnosis

The pooled results of two studies suggested a possible association between dialysis at diagnosis and poor renal outcomes during long-term follow-up (HR = 12.18, 95% *CI* 2.33–63.68).

###### Disease course

Three studies evaluated the association between disease course and poor renal outcomes, but no clear association was observed.

###### eGFR

Five studies evaluated the impact of eGFR as a continuous variable on poor renal outcomes. Three studies found that baseline eGFR may be associated with fewer poor renal outcomes within 1–2 years of follow-up (results of 2 studies: OR = 0.99, 95% *CI* 0.98–1.00; result of 1 study: HR = 0.98, 95% *CI* 0.97–1.00). The other two studies, with follow-up within 1 year and over 2 years, reported similar inverse associations (OR = 0.34, 95% *CI* 0.13–0.88; OR = 0.98, 95% *CI* 0.96–1.00).

Four studies evaluated the association between reduced eGFR (<60 ml/min/1.73m²) and poor renal outcomes. The results suggested that reduced eGFR may be associated with poor long-term renal outcomes (results of 3 studies: HR = 2.86, 95% *CI* 1.18–6.98; result of 1 study: OR = 11.80, 95% *CI* 2.22–62.73).

###### Hematological involvement

Three studies suggested that baseline hematological involvement may be associated with poor renal outcomes. This potential association was observed both within 1–2 years (HR = 3.09, 95% *CI* 1.23–7.75) and in long-term follow-up (OR = 4.47, 95% *CI* 2.33–8.59; HR = 3.56, 95% *CI* 0.86–14.74).

The pooled results of two studies indicated that a higher baseline hemoglobin level likely reduced the risk of poor renal outcomes within 1–2 years. (OR = 0.98, 95% *CI* 0.97–1.00).

###### Hypertension at diagnosis

One study found that baseline hypertension may be associated with poor renal outcomes during the 1–2-year follow-up period (OR = 8.74, 95% *CI* 1.19–64.19). Similarly, the pooled results of two studies suggested that baseline hypertension was likely associated with poor long-term renal outcomes (OR = 7.61, 95% *CI* 1.76–32.88). In contrast, the pooled results of two other long-term studies did not show a clear association between baseline hypertension and poor renal outcomes (HR = 0.82, 95% *CI* 0.24–2.74).

###### Scr at baseline

Six studies examined the association between baseline Scr levels and long-term poor renal outcomes. The pooled results of three studies suggested that increased baseline Scr may be associated with poor renal outcomes (HR = 3.81, 95% *CI* 1.53–9.48), despite heterogeneity in the specific definitions of “increased Scr” across these studies. The remaining three studies, which treated baseline Scr as a continuous variable, reported no clear association.

###### Kidney flare

The pooled results of three studies indicated that kidney flare may be associated with long-term poor renal outcomes (HR = 3.18, 95% *CI* 1.07–9.48). A similar potential association was also observed during the 1–2-year follow-up period (OR = 2.54, 95% *CI* 1.06–6.06).

###### Nephrotic range proteinuria

Two separate studies suggested that baseline nephrotic range proteinuria may be associated with poor renal outcomes during follow-up periods of 1 year and more than 2 years (OR = 3.38, 95% *CI* 1.60–7.16; OR = 3.86, 95% *CI* 1.33–11.18). However, another study found no clear evidence of an association between nephrotic-range proteinuria and poor renal outcomes within the 1–2-year follow-up period (HR = 3.52, 95% *CI* 0.44–28.16).

###### Nervous-system involvement

One study found that baseline neurological involvement may be associated with long-term poor renal outcomes (OR = 4.24, 95% *CI* 1.53–11.74), whereas another study evaluating the same long-term outcome found no clear association (HR = 1.18, 95% *CI* 0.13–10.71).

###### Renal non-remission at 6 months

One study found that failure to achieve renal remission within 6 months may be associated with disease progression to ESRD within 1–2 years of follow-up (OR = 9.24, 95% *CI* 1.26–67.76), but it was not clearly associated with mortality (HR = 2.20, 95% *CI* 0.47–10.30). The pooled results of three studies suggested that failure to achieve renal remission within 6 months may be associated with long-term poor renal outcomes (HR = 7.24, 95% *CI* 3.12–16.79).

###### Renal non-remission at 12 months

The pooled results of four studies suggested that failure to achieve renal remission within 12 months may be associated with long-term poor renal outcomes (HR = 3.92, 95% *CI* 1.98–7.78).

###### SLEDAI score

Two studies suggested that higher baseline SLEDAI scores may be associated with poor renal outcomes at 1-year follow-up and within 1–2 years of follow-up (OR = 2.59, 95% *CI* 1.26–5.34; OR = 2.18, 95% *CI* 1.72–2.77). However, two other studies did not identify a clear association between baseline SLEDAI scores and long-term poor renal outcomes (OR = 0.97, 95% *CI* 0.89–1.06; HR = 1.09, 95% *CI* 0.99–1.20).

##### Medication

###### Mycophenolic acid concentration-time curve area 0–12 h (MPA-AUC_0-12_h)

One study reported that higher MPA-AUC_0-12h_ was likely associated with better renal outcomes during 1-year follow-up (OR = 0.87, 95% CI 0.79–0.95). Similarly, another study suggested that MPA-AUC_0-12h_ may be inversely associated with long-term poor renal outcomes (HR = 0.95, 95% *CI* 0.92–0.98).

###### Treatment non-compliance

One study suggested that patients with poor treatment compliance had a higher risk of long-term poor renal outcomes (OR = 6.43, 95% CI: 2.23–18.57). The pooled results of two other studies evaluating long-term poor renal outcomes did not show a clear association (HR = 1.92, 95% *CI* 0.93–3.94).

### 3.3 Outcome-specific prognostic associations with poor renal outcomes

Outcome-specific prognostic associations were further evaluated to explore whether prognostic associations differed across specific renal outcome categories. All studies with ≤1 year of follow-up evaluated renal non-remission; therefore, no further outcome-specific analysis was required. Among studies with 1–2 years of follow-up, outcome-specific meta-analysis was feasible only for biopsy class III (versus other classes) in relation to renal non-remission, with the pooled estimate showing no clear association. Among the 22 studies with >2 years of follow-up, 10 evaluated CKD and/or ESRD, 3 mortality alone, 2 renal non-remission, and 7 composite outcomes including mortality, CKD, and ESRD. No pooled analysis was feasible for mortality alone or renal non-remission. Male sex and increased serum creatinine were associated with CKD and/or ESRD, whereas renal non-remission at 6 or 12 months was associated with the composite outcome.

### 3.4 GRADE of frequently evaluated prognostic factors

**Figure 8** summarizes the certainty of evidence for 17 frequently evaluated prognostic factors, including 1 demographic factor, 15 disease characteristics-related factors, and 1 medication-related factor. There is no high-quality evidence, and most of the prognostic factors are assessed as having moderate or low quality of evidence. Only one risk factor was identified at the 1-year follow-up and one at the 1–2-year follow-up, whereas nine risk factors were associated with long-term prognosis. A detailed assessment is provided in **Supplementary Table S6**.

**Figure 8:**
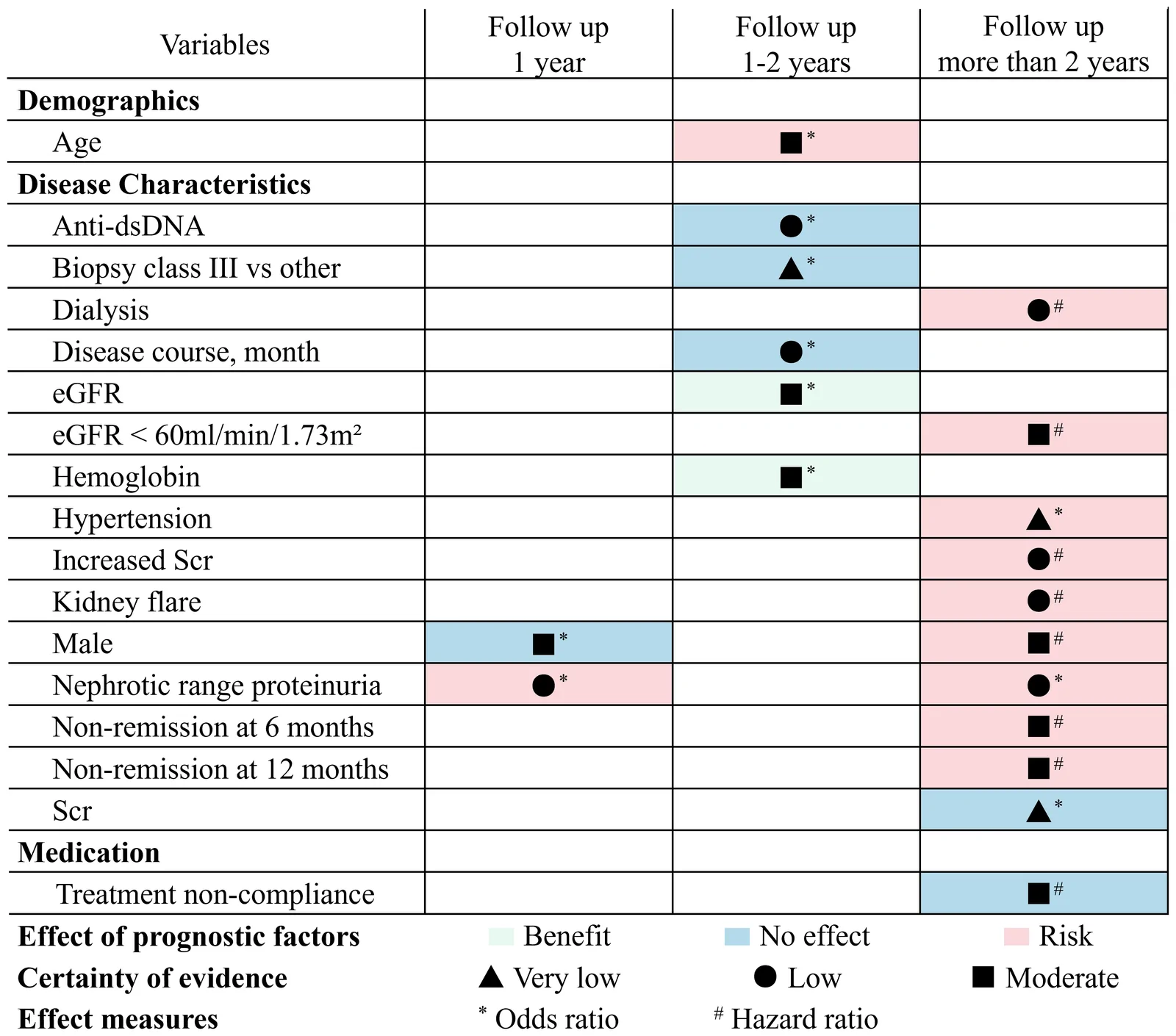
Summary of frequently evaluated prognostic factors for poor renal outcomes. Anti-dsDNA: anti-double-stranded DNA; eGFR: estimated glomerular filtration rate; Scr: serum creatinine.

### 3.5 Sensitivity analysis

In the first sensitivity analysis, a total of 3 studies were excluded (**Supplementary Table S4**). These included 1 study that defined renal non-remission as the need for intensified treatment after 1 year of follow-up, and 2 studies that used renal non-remission as the outcome measure after 2 years of follow-up. Regarding the frequently evaluated prognostic factors, no differences were found in the results, except for treatment compliance, which was excluded due to the inability to conduct a combined analysis.

The second sensitivity analysis excluded 23 studies with a high risk of bias (**Supplementary Table S5**). The analysis indicated that among the 17 prognostic factors originally identified, 3 were not confirmed as frequently evaluated prognostic factors: nephrotic range proteinuria, increased Scr, and Scr level.

## 4 Discussion

This is the first systematic review and meta-analysis to synthesize prognostic associations for poor renal outcomes in cLN across different follow-up duration categories. Based on 47 studies, 17 factors with available prognostic evidence were evaluated, and the certainty of evidence was assessed using GRADE. Nephrotic-range proteinuria was associated with poor renal outcomes within 1 year; older age, lower eGFR, and lower hemoglobin within 1–2 years; and dialysis at diagnosis, eGFR <60 mL/min/1.73 m², hypertension, kidney flare, male sex, and renal non-remission at 6 or 12 months among studies with >2 years of follow-up. However, nephrotic-range proteinuria and increased serum creatinine showed limited robustness after excluding studies at high risk of bias. The certainty of evidence ranged from low to moderate. However, these findings were derived from separate OR- and HR-based analyses. Because ORs estimate the odds of an outcome within a specified period, whereas HRs incorporate the timing of events, the two measures were interpreted separately and were not considered directly comparable. Higher pooled proportions of CKD, ESRD, and mortality were observed in studies with longer follow-up durations, whereas renal non-remission did not show a consistent pattern across follow-up duration categories.

Compared with the previous LN review (17), both studies identified Scr, eGFR, and proteinuria as important prognostic indicators. However, the previous review mainly highlighted serological and histological factors, including C3, anti-dsDNA, anti-C1q, biopsy class, and chronic lesions, whereas our pediatric-specific analysis identified dialysis, hypertension, lower hemoglobin, male sex, kidney flare, and renal non-remission at 6 or 12 months. These differences suggest that prognostic assessment in cLN may place greater emphasis on the severity of kidney dysfunction and early treatment response.

Our findings align with established knowledge: children with nephrotic-range proteinuria, elevated Scr, low eGFR, or requiring dialysis face a higher risk of poor renal outcomes.

Our findings were generally consistent with previous evidence: children with nephrotic-range proteinuria, elevated Scr, low eGFR, or requiring dialysis may have a higher likelihood of poor renal outcomes, reflecting greater baseline renal injury (25). Hypertension at diagnosis may be associated with poorer renal outcomes, as reported in general kidney disease populations (26, 27), likely through mechanisms including glomerulosclerosis and ischemic injury. Low hemoglobin was likely associated with poor renal outcomes, consistent with a pediatric LN cohort of 428 children in which patients without stable renal remission were less likely to have normal hemoglobin levels (28). Evidence from CKD populations also suggests reduced hemoglobin impairs renal oxygen delivery (29, 30). However, a J-shaped association between hemoglobin and CKD risk has also been reported (31), emphasizing the need for optimal hemoglobin levels. Older age at diagnosis showed an association with poor renal outcomes, possibly due to adolescent sex-hormone immune dysregulation (32) and poorer treatment compliance (33). Male sex may be associated with worse outcomes in our analyses, matching adult LN meta-analyses (34) and animal models (35). However, the specific mechanisms underlying this gender difference in kidney diseases remain unclear and may be attributed to hormonal and lifestyle factors (36). Failure to achieve short-term renal remission may indicate a higher likelihood of subsequent poor renal outcomes, highlighting the potential importance of early identification of non-responders.

Several factors not showing sufficient evidence of association in this review still warrant further investigation. Although SLEDAI, anti-dsDNA, and biopsy class did not show clear associations with poor renal outcomes in pooled analyses, they are typically considered associated with the progression of kidney damage (37, 38). Other immune-inflammatory indicators, including lymphocyte subsets, interleukins, and neutrophil-to-lymphocyte ratio, also suggested potential prognostic value. Notably, prolonged PM2.5 exposure and pulmonary infection were linked to poorer outcomes, possibly via systemic inflammation (39), and treatment non-adherence has been linked to renal deterioration in prior studies (40). However, these latter associations are derived from limited evidence and should be interpreted as hypothesis-generating observations, given potential biases from small sample sizes, population heterogeneity, and inadequate confounder adjustment. Future studies incorporating repeated measurements and time-dependent analytical methods are needed to clarify their prognostic roles in cLN.

Prognostic associations differed across studies categorized by follow-up duration. Most studies (n=27) focused on long-term outcomes (>2 years). Longer follow-up, with more cumulative events, allows the assessment of not only acute indicators but also factors associated with chronicity and cumulative damage, such as chronic inflammation and long-term environmental exposures. Future research should systematically track changes in each factor over time to develop risk stratification systems applicable to different disease stages. In addition to follow-up duration, the clinical interpretation of prognostic associations was also influenced by the specific renal endpoint assessed. Outcome-specific analyses demonstrated that prognostic associations differed across outcome categories, indicating that the clinical relevance of individual factors may depend partly on the specific endpoint assessed. Therefore, associations derived from pooled analyses of heterogeneous outcomes should not be interpreted as applying equally to each specific endpoint.

The primary goal of LN management is to preserve kidney function and reduce CKD, ESRD, and mortality (14). Our pooled proportion analyses showed that studies with longer follow-up durations reported higher proportions of CKD, ESRD, and mortality, with pooled estimates approaching 10% in the longer follow-up categories. This proportion was comparable to or lower than that reported in previous studies (41, 42), but still highlights the need for long-term management. A dual-target strategy addressing both immune activity and non-immune risk factors should be considered. This includes exploring emerging immunotherapies while managing modifiable factors (35, 43). However, dynamic risk-stratification models incorporating longitudinal clinical information remain limited, though models incorporating repeated measures have shown better predictive performance than static ones in other kidney diseases (44, 45). Future studies should therefore prioritize longitudinal prognostic assessment and the development of dynamic prediction models to support individualized management of cLN.

Rigorous critical appraisal is essential in medical reviews because the reliability of evidence synthesis depends on the methodological quality of the included studies (46). In this review, nearly half of the studies were rated as having a high risk of bias. First, covariate selection varied considerably and was often inadequately justified. Some studies relied solely on univariable analyses, which may omit important confounders or retain chance-associated variables, increasing the risk of false-positive and false-negative findings (47). Because adjustment sets also differed across studies, pooled multivariable estimates may not represent uniform independent effects and remain susceptible to residual confounding. Second, none of the studies adequately addressed time-varying confounding, such as changes in glucocorticoid dosage, which may have biased effect estimates (48, 49). Third, several studies reported dropout rates exceeding 10%, yet none used methods such as inverse probability weighting or multiple imputation to address attrition. When dropout is related to exposures or outcomes, this may reduce cohort representativeness and bias the estimated associations (50, 51). Finally, incomplete or erroneous reporting of variables included in multivariable analyses may have introduced selective reporting bias and reduced the reliability of both the original findings and the subsequent evidence synthesis (19).

The main strength of this study is its comprehensive synthesis of prognostic factors associated with poor renal outcomes in cLN through a systematic literature search, with outcome proportions summarized across different follow-up duration categories. However, this study has several limitations. Firstly, studies that only conducted univariate analysis were excluded, which may have enhanced the robustness of the results but limited the diversity of the prognostic factor spectrum. Secondly, although outcome-specific analyses were performed to address heterogeneity in outcome definitions, differences in the clinical meaning, measurement, and diagnostic definitions of renal involvement across original studies may still affect the interpretation of pooled results. Thirdly, follow-up duration was categorized at the study level rather than derived from patient-level longitudinal analyses. Therefore, differences across follow-up categories may partly reflect differences in study populations and methodologies rather than true temporal changes in prognostic effects. Finally, pooled proportions of renal outcomes were derived only from studies reporting prognostic factors and may not be fully generalizable to the broader cLN population due to potential differences in patient selection and clinical characteristics.

## 5 Conclusion

This systematic review and meta-analysis identified 17 factors with available prognostic evidence, including 1 demographic factor, 15 disease characteristics-related factors, and 1 medication-related factor. Prognostic associations differed across studies categorized by follow-up duration, outcome definitions, and effect measures; however, these findings should be interpreted cautiously because follow-up duration was categorized at the study level rather than derived from patient-level longitudinal analyses, and heterogeneous outcomes and effect measures may limit direct comparisons. Higher pooled proportions of CKD, ESRD, and mortality were observed among studies with longer follow-up durations, highlighting the need for continued renal monitoring. However, most included studies had methodological limitations, and the certainty of evidence was generally moderate or low; thus, these associations should be considered provisional rather than definitive and should not be used in isolation to guide clinical risk stratification. Future studies should adopt standardized outcome definitions, consistent confounder adjustment, and appropriate time-to-event and longitudinal methods to support the development of reliable, dynamic risk-stratification strategies for children with LN.

## Data Availability

The original contributions presented in the study are included in the article/**Supplementary Material**. Further inquiries can be directed to the corresponding authors.
