# Supplementary material for: Time-stratified prognostic factors for poor renal outcomes in childhood-onset lupus nephritis: a systematic review and meta-analysis
Source: Front Immunol. 2026 Sep 25;17:1939149. doi: 10.3389/fimmu.2026.1939149 (PMC13650986; doi:10.3389/fimmu.2026.1939149)
Supplement: Supplementary file 1 [file Supplementaryfile1.docx]

# Supplementary Table S1: PRISMA Checklist

| **Section and Topic** | **Item #** | **Checklist item** | **Location where item is reported** |
| --- | --- | --- | --- |
| **TITLE** | | |  |
| Title | 1 | Identify the report as a systematic review. | Title |
| **ABSTRACT** | | |  |
| Abstract | 2 | See the PRISMA 2020 for Abstracts checklist. | Abstract |
| **INTRODUCTION** | | |  |
| Rationale | 3 | Describe the rationale for the review in the context of existing knowledge. | Introduction |
| Objectives | 4 | Provide an explicit statement of the objective(s) or question(s) the review addresses. | Introduction |
| **METHODS** | | |  |
| Eligibility criteria | 5 | Specify the inclusion and exclusion criteria for the review and how studies were grouped for the syntheses. | Method-2.2 Study Selection |
| Information sources | 6 | Specify all databases, registers, websites, organisations, reference lists and other sources searched or consulted to identify studies. Specify the date when each source was last searched or consulted. | Method-2.1 Searching Strategy |
| Search strategy | 7 | Present the full search strategies for all databases, registers and websites, including any filters and limits used. | Method-2.1 Searching Strategy |
| Selection process | 8 | Specify the methods used to decide whether a study met the inclusion criteria of the review, including how many reviewers screened each record and each report retrieved, whether they worked independently, and if applicable, details of automation tools used in the process. | Method-2.2 Study Selection |
| Data collection process | 9 | Specify the methods used to collect data from reports, including how many reviewers collected data from each report, whether they worked independently, any processes for obtaining or confirming data from study investigators, and if applicable, details of automation tools used in the process. | Method-2.3 Data Extraction and Quality Assessment |
| Data items | 10a | List and define all outcomes for which data were sought. Specify whether all results that were compatible with each outcome domain in each study were sought (e.g. for all measures, time points, analyses), and if not, the methods used to decide which results to collect. | Method-2.3 Data Extraction and Quality Assessment, 2.4 Data Analysis |
|  | 10b | List and define all other variables for which data were sought (e.g. participant and intervention characteristics, funding sources). Describe any assumptions made about any missing or unclear information. | Method-2.3 Data Extraction and Quality Assessment, 2.4 Data Analysis |
| Study risk of bias assessment | 11 | Specify the methods used to assess risk of bias in the included studies, including details of the tool(s) used, how many reviewers assessed each study and whether they worked independently, and if applicable, details of automation tools used in the process. | Method-2.3 Data Extraction and Quality Assessment |
| Effect measures | 12 | Specify for each outcome the effect measure(s) (e.g. risk ratio, mean difference) used in the synthesis or presentation of results. | Method-2.4 Data Analysis |
| Synthesis methods | 13a | Describe the processes used to decide which studies were eligible for each synthesis (e.g. tabulating the study intervention characteristics and comparing against the planned groups for each synthesis (item #5)). | Method-2.4 Data Analysis |
|  | 13b | Describe any methods required to prepare the data for presentation or synthesis, such as handling of missing summary statistics, or data conversions. | Method-2.4 Data Analysis |
|  | 13c | Describe any methods used to tabulate or visually display results of individual studies and syntheses. | Method-2.4 Data Analysis |
|  | 13d | Describe any methods used to synthesize results and provide a rationale for the choice(s). If meta-analysis was performed, describe the model(s), method(s) to identify the presence and extent of statistical heterogeneity, and software package(s) used. | Method-2.4 Data Analysis |
|  | 13e | Describe any methods used to explore possible causes of heterogeneity among study results (e.g. subgroup analysis, meta-regression). | Method-2.4 Data Analysis |
|  | 13f | Describe any sensitivity analyses conducted to assess robustness of the synthesized results. | Method-2.6 Sensitivity Analysis |
| Reporting bias assessment | 14 | Describe any methods used to assess risk of bias due to missing results in a synthesis (arising from reporting biases). | Method-2.4 Data Analysis |
| Certainty assessment | 15 | Describe any methods used to assess certainty (or confidence) in the body of evidence for an outcome. | Method-2.5 Certainty of The Evidence Assessment |
| **RESULTS** | | |  |
| Study selection | 16a | Describe the results of the search and selection process, from the number of records identified in the search to the number of studies included in the review, ideally using a flow diagram. | Results-3.1 Study Characteristics and Quality Assessment |
|  | 16b | Cite studies that might appear to meet the inclusion criteria, but which were excluded, and explain why they were excluded. | Supplementary materials |
| Study characteristics | 17 | Cite each included study and present its characteristics. | Supplementary materials |
| Risk of bias in studies | 18 | Present assessments of risk of bias for each included study. | Results-3.1 Study Characteristics and Quality Assessment |
| Results of individual studies | 19 | For all outcomes, present, for each study: (a) summary statistics for each group (where appropriate) and (b) an effect estimate and its precision (e.g. confidence/credible interval), ideally using structured tables or plots. | Results-3.2 Overall prognostic associations with poor renal outcomes, 3.3 Outcome-specific prognostic associations with poor renal outcomes |
| Results of syntheses | 20a | For each synthesis, briefly summarise the characteristics and risk of bias among contributing studies. | Results-3.2 Overall prognostic associations with poor renal outcomes, 3.3 Outcome-specific prognostic associations with poor renal outcomes |
|  | 20b | Present results of all statistical syntheses conducted. If meta-analysis was done, present for each the summary estimate and its precision (e.g. confidence/credible interval) and measures of statistical heterogeneity. If comparing groups, describe the direction of the effect. | Results-3.2 Overall prognostic associations with poor renal outcomes, 3.3 Outcome-specific prognostic associations with poor renal outcomes |
|  | 20c | Present results of all investigations of possible causes of heterogeneity among study results. | Results-3.5 Sensitivity Analysis |
|  | 20d | Present results of all sensitivity analyses conducted to assess the robustness of the synthesized results. | Results-3.5 Sensitivity Analysis |
| Reporting biases | 21 | Present assessments of risk of bias due to missing results (arising from reporting biases) for each synthesis assessed. | NA |
| Certainty of evidence | 22 | Present assessments of certainty (or confidence) in the body of evidence for each outcome assessed. | Results-3.4 GRADE of Frequently Evaluated Prognostic Factors |
| **DISCUSSION** | | |  |
| Discussion | 23a | Provide a general interpretation of the results in the context of other evidence. | Discussion |
|  | 23b | Discuss any limitations of the evidence included in the review. | Discussion |
|  | 23c | Discuss any limitations of the review processes used. | Discussion |
|  | 23d | Discuss implications of the results for practice, policy, and future research. | Discussion |
| **OTHER INFORMATION** | | |  |
| Registration and protocol | 24a | Provide registration information for the review, including register name and registration number, or state that the review was not registered. | Method |
|  | 24b | Indicate where the review protocol can be accessed, or state that a protocol was not prepared. | Method |
|  | 24c | Describe and explain any amendments to information provided at registration or in the protocol. | NA |
| Support | 25 | Describe sources of financial or non-financial support for the review, and the role of the funders or sponsors in the review. | Declarations |
| Competing interests | 26 | Declare any competing interests of review authors. | Declarations |
| Availability of data, code and other materials | 27 | Report which of the following are publicly available and where they can be found: template data collection forms; data extracted from included studies; data used for all analyses; analytic code; any other materials used in the review. | Declarations |

*From:* Page MJ, McKenzie JE, Bossuyt PM, Boutron I, Hoffmann TC, Mulrow CD, et al. The PRISMA 2020 statement: an updated guideline for reporting systematic reviews. BMJ 2021;372:n71. doi: 10.1136/bmj.n71

# Supplementary Table S2: Search Strategy

**PubMed**

#1 ((((((((Lupus Nephritis[MeSH Terms]) OR (Lupus Nephritis[Title/Abstract])) OR (Glomerulonephritis, Lupus[Title/Abstract])) OR (Glomerulonephritides, Lupus[Title/Abstract])) OR (Lupus Glomerulonephritides[Title/Abstract])) OR (Lupus Glomerulonephritis[Title/Abstract])) OR (Nephritis, Lupus[Title/Abstract])) OR (Lupus Nephritides[Title/Abstract])) OR (Nephritides, Lupus[Title/Abstract])

#2 (((((Child[MeSH Terms]) OR (Child*[Title/Abstract])) OR (Adolescent[MeSH Terms])) OR (Adolescen*[Title/Abstract])) OR (youth*[Title/Abstract])) OR (teen*[Title/Abstract])

#3 ((((((((((risk*[Title/Abstract]) OR (variable*[Title/Abstract])) OR (factor*[Title/Abstract])) OR (predict*[Title/Abstract])) OR (Prognosis[MeSH Terms])) OR (Prognos*[Title/Abstract])) OR (Treatment Outcome[MeSH Terms])) OR (efficacy[Title/Abstract])) OR (effec*[Title/Abstract])) OR (treatment outcome[Title/Abstract])) OR (biomarker*[Title/Abstract])

#4 #1 AND #2 AND #3

**Embase**

#1 'lupus erythematosus nephritis'/exp

#2 'glomerulonephritis, lupoid':ab,ti OR 'lupoid nephritis':ab,ti OR 'lupus erythematosus, nephritis':ab,ti OR 'lupus glomerulonephritis':ab,ti OR 'lupus kidney':ab,ti OR 'lupus nephritis':ab,ti OR 'lupus nephropathy':ab,ti OR 'nephritis, lupus erythematosus':ab,ti OR 'nephritis, systemic lupus erythematosus':ab,ti OR 'systemic lupus erythematosis, nephritis':ab,ti OR 'lupus erythematosus nephritis':ab,ti

#3 #1 OR #2

#4 'child'/exp

#5 'adolescent'/exp

#6 child*:ab,ti OR adolescen*:ab,ti OR youth*:ab,ti OR teen*:ab,ti

#7 #4 OR #5 OR #6

#8 'prognosis'/exp

#9 'treatment outcome'/exp

#10 prognos*:ab,ti OR efficacy:ab,ti OR effect*:ab,ti OR 'treatment outcome':ab,ti OR risk*:ab,ti OR variable*:ab,ti OR factor*:ab,ti OR predict*:ab,ti OR biomarker*:ab,ti

#11 #8 OR #9 OR #10

#12 #3 AND #7 AND #11

**Scopus**

#1 TITLE-ABS-KEY ( "Lupus Nephritis" OR "Glomerulonephritis, Lupus" OR "Lupus Glomerulonephritides" OR "Lupus Glomerulonephritis" OR "Lupus Nephritides" )

#2 TITLE-ABS-KEY ( Child* OR Adolescen* OR youth* OR teen* )

#3 TITLE-ABS-KEY ( risk* OR variable* OR factor* OR predict* OR Prognos* OR efficacy OR effect* or "treatment Outcome" OR biomarker*)

#4 #1 AND #2 AND #3

**China National Knowledge Infrastructure: CNKI**

(TKA = 'Lupus Nephritis') AND (TKA = 'child*' OR TKA = 'adolescen*') AND (TKA = 'risk*' OR TKA = 'predict*' OR TKA = 'prognos*' OR TKA = 'effect' OR TKA = 'biomarker*')

**Wan Fang**

Abstract:(Lupus Nephritis) and (Abstract:(child*) or Abstract:(adolescen*)) and (Abstract:(risk*) or Abstract:(predict*) or Abstract:(prognos*) or Abstract:(effect) or Abstract:(biomarker*))

**VIP**

R=(Lupus Nephritis) AND R=(child* OR adolescen*) AND R=(risk* OR predict* OR prognos* OR effect OR biomarker*)

**Sino Med**

" Lupus Nephritis"[Abstract] AND ("child*"[Abstract] OR "adolescen*"[Abstract]) AND ("risk*"[Abstract] OR "predict*"[Abstract] OR "prognos*"[Abstract] OR "effect"[Abstract] OR "biomarker*"[Abstract])

# Supplementary Table S3: Characteristics of Included Studies

| Author year | Country | Study Design | Popula--tion | Age (y) at LN diagnosis | Kidney Biopsy  class n (%) | Follow up | Main findings | Analysis methods |
| --- | --- | --- | --- | --- | --- | --- | --- | --- |
| Asis 2025(1) | Philippines | RCS | 128 | 14 (mean) | III 8 (6.3) | 1.8 y (median) | Hypertension at diagnosis and failure to achieve remission at 6 months were independent risk factors for stage 5 CKD. | MLR/MCR |
| Balbi 2022(2) | Brazil | PCS | 60 | ＜18 | No information | 5.8 mo (median) | Low hydroxychloroquine blood levels were an independent risk factor for kidney flare. | MLR |
| Chan 2022(3) | China | RCS | 92 | 13.7 (mean) | IV 43 (46.7) | 10.3 y (median) | The need for dialysis at diagnosis, failure to achieve remission at 12 months, and multiple episodes of kidney flare were independent risk factors for poor prognosis. | MCR |
| Chan 2024(4) | China | RCS | 95 | 13.6 (mean) | IV 44 (46.3) | 10.2 y (mean) | Diagnosis age ≤ 13.1 years, maintenance treatment with azathioprine, and failure to achieve remission at 12 months were independent risk factors for kidney flare. | MCR |
| Chen 2025(5) | China | RCS | 48 | 11.0 (mean) | IV/V 21 (43.8) | 1 y after treatment | High MPA-AUC_0-12h_ was an independent protective factor for achieving renal remission. | MLR |
| Cheng 2021(6) | China | RCS | 343 | 13.0 (median) | IV 135 (39.4) | 5 y (median) | Increased baseline Scr and kidney flare were independent risk factors for ESRD. | MCR |
| Demir 2022(7) | Turkey | RCS | 53 | 13.1 (median) | IV 29 (54.7) | 4 y (median) | Male sex, the need for dialysis at diagnosis, and failure to achieve remission at 6 and 12 months were independent risk factors for poor renal outcome. | MCR |
| Dubrasquet 2021(8) | France | RCS | 27 | 12.4 (median) | IV 12 (44.4) | > 6 mo | An MPA-AUC_0-12h_ > 45 mg·h/L was independently associated with treatment response. | MLR |
| Gao 2001(9) | China | RCS | 74 | 10.4 (mean) | IV 25 (53.0) | 3.6 y (mean) | Baseline hypertension, heavy proteinuria, marked hematuria, persistent anemia, and increased baseline Scr were identified as independent predictors of progression to renal failure. | MLR |
| Han 2025(10) | China | RCS | 75 | ＜18 | IV 44 (58.7) | 6 mo after treatment | Edema at diagnosis, positive anti-dsDNA antibody, and cyclophosphamide treatment were independent risk factors for failure to remission. | MLR |
| He 2024(11) | China | RCS | 342 | 13.4 (median) | IV 208 (60.8) | 8.1 y (median) | PM2.5 and its components were independently associated with an increased risk of mortality and renal failure. | MCR |
| Jiao 2022(12) | China | RCS | 100 | 10.3 (mean) | No information | 2 y after treatment | 24-hour high urinary protein, increased serum TNF-R1 and IGFBP-2, and decreased serum CFH were independent risk factors for poor prognosis. | MCR |
| Lama 2025(13) | India | RCS/PCS | 75 | 11.2 (median) | IV 34 (45.3) | 2.3 y (median) | Baseline RPGN, non-remission at 6 months, and kidney flare were independent risk factors for poor prognosis. | MCR |
| Li 2022(14) | China | RCS | 64 | ＜18 | IV 64 (100) | 6 mo after treatment | ECHC < 50% and increased baseline 24-hour urinary protein were independent predictors of complete remission. | MLR |
| Li 2023(15) | China | RCS | 61 | ＜18 | IV 34 (55.7) | 2.8 y (median) | Low MPA-AUC_0-12h_, increased IgG, and NLR were independent risk factors for kidney flare. | MCR |
| Liang 2004(16) | China | RCS | 63 | ≤14 | No information | 6.5 y (mean) | Delayed diagnosis, SLEDAI > 20, increased Scr, heavy proteinuria, and central nervous system involvement were identified as independent predictors of renal dysfunction or mortality. | MCR |
| Liu 2022(17) | China | PCS | 70 | 12.3 (mean) | No information | 2.2 y (mean) | Increased serum HB-EGF and HIF-1α expression were independent risk factors for ESRD or mortality. | MLR |
| Liu 2024(18) | China | CCS | 138 | 14.9 (mean) | IV 66 (47.8) | 8.6 y (median) | The presence of TMA was an independent risk factor for ESRD. | MLR |
| Lu 2024(19) | China | RCS | 47 | 10.9 (mean) | IV 22 (46.8) | 4.6 y (mean) | Increased 24-hour urinary protein, decreased CD3+ cells, and increased CD19+ cells were independent risk factors for CKD or mortality. | MCR |
| Luan 2013(20) | China | RCS | 67 | 12.2 (mean) | IV 41 (61.2) | ＞2 y | Male sex, increased Scr, and low serum complement C4 were independent risk factors for reduced kidney RSR. | MCR |
| Luan 2013(21) | China | RCS | 57 | 10.4 (mean) | IV 57 (100) | ＞1 y | Decreased Ccr, failure to achieve remission, and kidney flare were independent risk factors for chronic renal failure. | MLR |
| Luan 2014(22) | China | RCS | 67 | 12.2 (mean) | IV 41 (61.2) | ＞2 y | Chronic index > 3 was an independent risk factor for reduced RSR. | MCR |
| Mutiis 2023(23) | Italy | RCS | 351 | 12.4 (median) | IV 158 (45.0) | 2 y after treatment | High levels of hemoglobin and eGFR at diagnosis, along with biopsy class III, were independent predictors of stable complete remission. | MLR |
| Park 2025(24) | Korea | RCS | 216 | 13.2 (mean) | IV 138 (63.9) | 7.8 y (mean) | Male sex and failure to achieve remission at 12 months were independent risk factors for advanced CKD. | MCR |
| Patrizi 2024(25) | USA | RCS | 74 | 12.8 (mean) | III or IV 52 (70.3) | 1 y after treatment | An extrarenal domain count ≥ 6 and a disease activity score ≥ 30 were not independent predictors of failure to achieve a complete renal response. | MLR |
| Qiu 2022(26) | China | RCS | 137 | 11.0 (mean) | IV 21 (15.3) | 2.3 y (median) | Poor medication adherence, baseline neurological involvement, baseline hypertension, and reduced eGFR were independent risk factors for poor renal prognosis. | MLR |
| Rianthavorn 2019(27) | Thailand | CCS | 179 | ＜18 | III 139 (77.7) | ＞1 y | NLR > 20, IFI, and high-dose prednisone were independent risk factors for infection-related mortality. | MLR |
| Ruggiero 2013(28) | Italy | RCS | 161 | ＜18 | IV 75 (46.6) | 8 y (mean) | Female sex, biopsy class IV, and delayed-onset SLE were independent risk factors for poor renal outcome, whereas older age at diagnosis and higher treatment intensity were independent protective factors. | MCR |
| Smith 2018(29) | UK | RCS | 64 | 14 (median) | IV 28 (43.8) | 1.4 y (median) | Older age at diagnosis, high baseline eGFR, and absence of hematologic involvement were independent predictors of proteinuria remission. | MCR |
| Smitherman 2023(30) | USA | RCS | 107 | 13.6 (mean) | IV 79 (73.8) | 1.4 y (median) | Male sex and older age at diagnosis were independent predictors of abnormal renal function. | MLR |
| Srivastava 2016(31) | India | RCS | 134 | 13.6 (mean) | IV 43 (30.6) | 6.8 y (mean) | Baseline Scr ≥ 1.5 mg/dL and disease-related damage accumulation were identified as independent predictors of mortality or progression to ESRD. | MLR |
| Stotter 2023(32) | USA | RCS | 59 | ＜16 | IV or IV/V 31 (52.5) | 3.9 y (mean) | Female sex and electrolyte disturbances were independent risk factors for ESRD. | MLR |
| Suhlrie 2020(33) | Germany | RCS/PCS | 79 | 13.7 (median) | IV 68 (86.1) | 1 y after treatment | Baseline nephrotic range proteinuria was an independent risk factor for failure to achieve complete remission. | MLR |
| Taheri 2011(34) | Iran | RCS | 60 | 10.5 (mean) | IV 42 (70.0) | 3.7 y (mean) | Concomitant pericarditis was an independent risk factor for mortality. | MCR |
| Vachvanichsanong 2009(35) | Thailand | RCS | 177 | 11.8 (mean) | IV 94 (53.1) | 3.9 y (median) | Male sex was an independent risk factor for mortality. | MCR |
| Wang 2024(36) | USA | RCS | 145 | 13.9 (mean) | IV 109 (75.2) | 1 y after treatment | Neither the EuroLupus nor the NIH cyclophosphamide regimen was identified as an independent predictor of renal remission. | MLR |
| Wisanuyotin 2022(37) | Korea | RCS | 113 | ＜18 | V 94 (83.2) | 6 mo after treatment | Baseline non-nephrotic range proteinuria and baseline high eGFR were independent predictors of renal remission. | MLR |
| Wu 2014(38) | China | PCS | 104 | 12.4 (mean) | IV 70 (67.3) | 7.2 y (mean) | Baseline SLEDAI > 20 and baseline eGFR < 60 mL/min/1.73m² were independent risk factors for mortality or ESRD. Early response at 6 months was an independent protective factor for poor outcome. | MCR |
| Wu 2016(39) | China | RCS | 65 | 12.6 (mean) | IV 36 (55.4) | 10.4 y (mean) | Increased serum IL-18 was an independent risk factor for mortality or ESRD. | MLR |
| Wu 2018(40) | China | RCS | 60 | 12.9 (median) | IV 43 (71.7) | 7.0 y (median) | Increased urinary clusterin-to-creatinine ratio was an independent risk factor for ESRD. | MCR |
| Xie 2020(41) | China | PCS | 68 | 9.8 (mean) | V 26 (38.2) | 2 y after treatment | Increased serum BAFF were identified as independent predictors of failure to achieve renal remission, progression to renal failure, or mortality. | MLR |
| Xing 2025(42) | China | PCS | 123 | 10.1 (mean) | IV 53 (43.1) | 1 y after treatment | Increased SLEDAI score, increased activity score, treatment non-adherence, and increased levels of serum CD62P and CD44 were independent risk factors for poor prognosis. | MCR |
| Yang 2024(43) | China | CCS | 120 | 10.6 (mean) | No information | 1 y after treatment | Increased SLEDAI score, increased levels of serum IL-6 and ESR, and a low CD4+/CD8+ ratio were independent risk factors for poor prognosis. | MLR |
| Yıldırım 2025(44) | Turkey | RCS | 103 | < 18 | IV 54 (52.4) | > 2 y | Baseline heavy proteinuria and increased baseline SLEDAI were independent risk factors for ESRD. | MCR |
| Zawavi 2024(45) | Malaysia | CCS | 65 | < 18 | IV 53 (81.5) | > 5 y | Degreased eGFR and biopsy class IV were independent risk factors for CKD. | MLR |
| Zhang 2023(46) | China | RCS | 191 | 14.9 (mean) | IV 47 (24.6) | 1.6 y (median) | eGFR ≤ 60 mL/min/1.73m², a-C1q ≥ 20 U/mL, glomerulosclerosis, and increased chronic index were independent risk factors for ESRD. Age > 12 years and eGFR > 90 mL/min/1.73m² were independent predictors of proteinuria remission. | MCR |
| Zhang 2024(47) | China | CCS | 222 | 14.7 (mean) | IV 82 (36.9) | 4.2 y (mean) | Concomitant renal TMA and renal interstitial fibrosis were independent risk factors for ESRD. | MCR |

Footnote: **a-C1q:** anti-complement 1q; **Anti-dsDNA:** anti-double-stranded DNA; **BAFF:** B-cell activating factor; **C4:** complement 4; **Ccr:** creatinine clearance rate; **CCS:** case-control study; **CFH:** complement factor H; **CKD:** chronic kidney disease; **ECHC:** endocapillary hypercellularity; **eGFR:** estimated glomerular filtration rate; **ESR:** erythrocyte sedimentation rate; **ESRD:** end-stage renal disease; **HB-EGF:** heparin binding epidermal growth factor; **HIF-1α:** hypoxia inducible factor-1α; **IFI:** invasive fungal infection; **IgG:** immunoglobulin G; **IGFBP-2:** Insulin-like growth factor binding protein-2; **IL-6:** interleukin-6; **IL-18:** interleukin-18; **MCR:** multivariate cox regression; **MLR:** multivariate logistic regression; **MPA-AUC:** mycophenolic acid-area under concentration-time curve; **NLR:** neutrophil-to-lymphocyte ratio; **NIH:** national institutes of health; **PCS:** Prospective cohort study; **PM 2.5:** particulate matter with an aerodynamic diameter ≤ 2.5µm; **RCS:** retrospective cohort study; **RPGN:** rapidly progressive glomerulonephritis; **RSR:** renal survival rate; **Scr:** serum creatinine; **SLE:** systemic lupus erythematosus; **SLEDAI:** systemic lupus erythematosus disease activity index; **TNF-R1:** soluble tumor necrosis factor receptor 1; **TMA:** thrombotic microangiopathy.

# Supplementary Table S4: Covariates and outcomes of included studies

| Author year | Covariates | Outcome definitions |
| --- | --- | --- |
| **Follow up 1 year** | | |
| Balbi 2022* | hydroxychloroquine blood levels, prednisone use, SLEDAI score, treatment adherence | SLEDAI score or glucocorticoid dose or intensive immunosuppressive therapy |
| Chen 2025 | anti-dsDNA, complement 3, complement 4, creatinine clearance, MPA-AUC_0-12h_, mycophenolate mofetil dose, tacrolimus trough concentration | UPCR (or 24-hour urine protein) or eGFR |
| Dubrasquet 2021 | age, biopsy class, MPA-AUC ≥ 45 mg h/L, sex,  time from mycophenolate mofetil initiation | eGFR and 24-hour urine protein and Hematuria |
| Han 2025 | age, albumin, anti-dsDNA (+), BUN, cyclophosphamide, edema (+), sex | UPCR or eGFR |
| Li 2022 | complement 3, D-dimer, ECHC > 50%, eGFR, GBM thickening, SLEDAI score, urine protein | 24-hour urine protein or (Scr and albumin) |
| Patrizi 2024 | disease course, number of extrarenal domains, race and ethnicity, insurance, sex, summary score < 30 | UPCR and Scr |
| Suhlrie 2020 | age < 12 years, eGFR, initial therapy regimen, nephrotic range proteinuria | UPCR or eGFR |
| Wang 2024 | cyclophosphamide regimen | UPCR or eGFR |
| Wisanuyotin 2022 | angiotensin-converting enzyme inhibitor use,  eGFR, hydroxychloroquine use, induction therapy, non-nephrotic range proteinuria, sex | UPCR and eGFR |
| Xing 2025 | 24-hour urine protein, activity score, albumin, biopsy class, CD44, CD62P, complement 3, complement 4, eGFR, ESR, hemoglobin, SLEDAI score, treatment adherence | Scr or eGFR or ESRD or mortality |
| Yang 2024 | age, CD4^+^/CD8^+^, ESR, IL-6, Sex, SLEDAI score | Scr or eGFR |
| **Follow up 1-2 years** | | |
| Asis 2025 | failure to achieve remission at 3 months, failure to achieve remission at 6 months, hypertension, nephrotic range proteinuria | ESRD or mortality |
| Jiao 2022 | 24-hour urine protein, anti-dsDNA, CFH, IGFBP-2, TNF-R1 | CKD or ESRD or mortality |
| Luan 2013 | Ccr, kidney flare, non-remission | CKD or ESRD |
| Mutiis 2023 | eGFR, albumin, anti-dsDNA, hemoglobin, UPCR | UPCR and (eGFR and Scr) |
| Rianthavorn 2019 | daily prednisolone dose, eGFR, hemoglobin, hospital acquired infection, invasive fungal infection, NLR > 20, pulmonary infection, urine protein excretion | mortality |
| Smith 2018 | age, eGFR, hematological involvement | UPCR or UACR |
| Smitherman 2023 | age, biopsy class, disease course, sex | eGFR or UPCR |
| Xie 2020 | acute index, age, anti-dsDNA, biopsy class, chronic index, complement 3, disease course, serum BAFF, sex, SLEDAI | CKD or ESRD or mortality |
| Zhang 2023 | a-C1q ≥ 20 U/ml, age > 12 years, chronic index, eGFR ≤ 60 ml/min/1.73m², eGFR > 90 ml/min/1.73m², glomerular sclerosis | ESRD or UPCR |
| **Follow up over 2 years** | | |
| Chan 2022 | dialysis, number of kidney flare, remission at 12 months, treatment nonadherence | CKD or ESRD or mortality |
| Chan 2024 | age ≤ 13.1 years, maintenance therapy, non-remission at 12 months, treatment nonadherence | CKD or ESRD or mortality |
| Cheng 2021 | increased Scr, kidney flare, SLEDAI score | ESRD |
| Demir 2022 | age < 12 years, anti-phospholipid (+), dialysis, eGFR ≤ 60 ml/min/1.73m², hypertension, hypoalbuminemia, increased Scr, kidney flare, neurological involvement, non-remission at 6 months, non-remission at 12 months, serositis, sex, SLEDAI score, thrombocytopenia | CKD or ESRD |
| Gao 2001 | age ≥ 10 years, hematuria (0~++), hemoglobin (≥ 105g/L), hypertension, nephrotic range proteinuria, sex | ESRD |
| He 2024 | age, biopsy class, eGFR, hypertension, nephrotic range proteinuria, PM 2.5, Sex, SLEDAI score | ESRD |
| Lama 2025 | non-remission at 6 months, RPGN, severe kidney flare | CKD or ESRD or mortality |
| Li 2023* | IgG, MPA-AUC_0-12h_, NLR | 24-hour urine protein or Scr |
| Liang 2004 | central nervous system involvement, disease course, increased Scr, nephrotic range proteinuria, SLEDAI score > 20 | Mortality |
| Liu 2022 | BUN, HB-EGF, HIF-1α, increased Scr | Scr or CKD or ESRD or mortality |
| Liu 2024 | eGFR ≤ 60 ml/min/1.73m², glomerular sclerosis, TMA | ESRD |
| Lu 2024 | 24-hour urine protein, CD3^+^, CD19^+^ | eGFR or Scr or mortality |
| Luan 2013 | complement 3, complement 4, sex | ESRD |
| Luan 2014 | chronic index, glomerular sclerosis | ESRD |
| Park 2025 | failure to achieve remission at 12 months, sex | CKD or ESRD |
| Qiu 2022 | anti-dsDNA (+), eGFR, hypertension, hypoproteinemia, nephrotic range proteinuria, nervous system involvement, Scr, SLEDAI score, treatment non-adherence | eGFR or 24-hour urine protein or UPCR or ESRD or mortality |
| Ruggiero 2013* | acute renal failure, age, biopsy class, delay-SLE, hypertension, treatment score | eGFR or 24-hour urine protein |
| Srivastava 2016 | age, biopsy class, damage accrual, disease course,  hypertension, increased Scr (≥ 1.5mg/dL), infection, kidney flare, low complement 3, nephrotic range proteinuria, non-remission at 12 months, SLEDAI score | ESRD or mortality |
| Stotter 2023 | active index, age, biopsy class, chronic index, electrolyte disturbance, interstitial fibrosis, sex, time to acute dialysis discontinuation, tubular atrophy | ESRD |
| Taheri 2011 | pericarditis | mortality |
| Vachvanichsanong 2009 | age (> 9-12 years, > 12-14 years, > 14 years), biopsy class, interval from SLE diagnosis to biopsy (1-3 months, > 3 months), sex | mortality |
| Wu 2014 | eGFR, remission at 6 months, SLEDAI score > 20 | ESRD or mortality |
| Wu 2016 | IL-18, Scr | ESRD or mortality |
| Wu 2018 | NGAL/creatinine, TFF3/creatinine, urinary clusterin/creatinine | ESRD |
| Yıldırım 2025 | ANA (+), anti-ds-DNA (+), anti-phospholipid antibody (+), age, biopsy class, BUN, C-reactive protein, complement 3, complement 4, creatinine, delay time to diagnosis, direct coombs (+), ESR, hypertension, plasmapheresis, proteinuria (> 0.5g/d), pulse corticosteroid treatment, rituximab treatment, Sex, SLEDAI score | ESRD |
| Zawavi 2024 | biopsy class, eGFR | CKD |
| Zhang 2024 | interstitial fibrosis, increased Scr (≥ 110µmol/L), TMA | ESKD |

Footnote: ***:** excluded in the first sensitive analysis; **a-C1q:** anti-complement 1q; **Anti-dsDNA:** anti-double-stranded DNA; **BAFF:** B-cell activating factor; **BUN:** blood urea nitrogen; **Ccr:** creatinine clearance rate; **CFH:** complement factor H; **ECHC:** endocapillary hypercellularity; **eGFR:** estimated glomerular filtration rate; **ESR:** erythrocyte sedimentation rate; **ESRD:** end-stage renal disease; **GBM:** glomerular basement membrane; **HB-EGF:** heparin binding epidermal growth factor, **HIF-1α:** hypoxia inducible factor-1α; **IGFBP-2:** Insulin-like growth factor binding protein-2; **IgG:** immunoglobulin G; **IL-6**: interleukin-6; **IL-18:** interleukin-18; **MPA-AUC:** mycophenolic acid-area under concentration-time curve; **NGAL:** neutrophil gelatinase-associated lipocalin; **NLR:** neutrophil-to-lymphocyte ratio; **PM 2.5:** particulate matter with an aerodynamic diameter ≤ 2.5µm; **RPGN:** rapidly progressive glomerulonephritis; **Scr:** serum creatinine; **SLE:** systemic lupus erythematosus; **SLEDAI:** systemic lupus erythematosus disease activity index; **TNF-R1:** soluble tumor necrosis factor receptor 1; **TMA:** thrombotic microangiopathy; **TFF3:** trefoil factor 3 ANA: anti-nuclear antibody; **UACR:** urine albumin creatinine ratio; **UPCR:** urine protein-to-creatinine ratio.

# Supplementary Table S5: Risk of bias assessment (QUIPS)

| . | Q1: Study Participation | Q2: Study Attrition | Q3: Prognostic Factor Measurement | Q4: Outcome Measurement | Q5: Study Confounding | Q6: Statistical Analysis and Reporting | Overall quality assessment |
| --- | --- | --- | --- | --- | --- | --- | --- |
| Asis 2025 | Low | Low | Low | Low | Low | Low | Low |
| Balbi 2022 | High | Low | Low | Low | Low | Moderate | High |
| Chan 2022 | Low | Low | Low | Low | Low | Low | Low |
| Chan 2024 | Low | Low | Moderate | Low | Low | Low | Low |
| Chen 2025 | High | High | Low | Moderate | Low | Low | High |
| Cheng 2021 | Low | Moderate | Low | Low | Moderate | Moderate | High |
| Demir 2022 | Low | Low | Moderate | Low | Low | Low | Low |
| Dubrasquet 2021 | High | High | Moderate | Moderate | Low | Moderate | High |
| Gao 2001 | Low | Moderate | Moderate | Low | Low | Low | Moderate |
| Han 2025 | Low | Moderate | Low | Low | Low | Low | Low |
| He 2024 | Low | Moderate | Low | Low | Low | Low | Low |
| Jiao 2022* | Low | Moderate | Low | Moderate | Moderate | High | High |
| Lama 2025 | Low | Low | Low | Low | Low | Moderate | Low |
| Li 2022 | Low | Moderate | Low | Low | Moderate | Low | Moderate |
| Li 2023 | High | Moderate | Low | Low | Low | Moderate | High |
| Liang 2004* | Low | Moderate | Moderate | Low | Low | High | High |
| Liu 2022* | Low | Moderate | Moderate | Moderate | Moderate | High | High |
| Liu 2024 | Low | Moderate | Low | Low | Moderate | Moderate | High |
| Lu 2024 | Low | Low | Low | Low | Moderate | Low | Low |
| Luan 2013 | Low | Moderate | Low | Moderate | Low | Moderate | High |
| Luan 2013 (2) | Low | Moderate | Moderate | Moderate | Low | Moderate | High |
| Luan 2014* | Low | Moderate | Moderate | Moderate | Moderate | High | High |
| Mutiis 2023 | Moderate | Moderate | Low | Low | Low | Low | Moderate |
| Park 2025 | Low | Moderate | Low | Low | Low | Moderate | Moderate |
| Patrizi 2024 | Low | Low | Low | Low | Low | Low | Low |
| Qiu 2022 | Low | High | Moderate | Low | Low | Low | High |
| Rianthavorn 2019 | Low | Moderate | Moderate | Low | Low | Low | Moderate |
| Ruggiero 2013 | Low | Moderate | Low | Low | Low | Low | Low |
| Smith 2018 | Low | Moderate | Low | Low | Low | Moderate | Moderate |
| Smitherman 2023 | Low | Moderate | Low | Low | Low | Low | Low |
| Srivastava 2016 | Low | Moderate | Moderate | Moderate | Low | Moderate | High |
| Stotter 2023 | Moderate | Moderate | Moderate | Low | Low | Low | High |
| Suhlrie 2020 | Moderate | Moderate | Low | Low | Low | Moderate | High |
| Taheri 2011 | Low | Moderate | Moderate | Low | Moderate | Moderate | High |
| Vachvanichsanong 2009 | Low | Low | Moderate | Low | Moderate | Low | Moderate |
| Wang 2024 | Moderate | Moderate | Low | Low | Low | Low | Moderate |
| Wisanuyotin 2022 | Low | Moderate | Moderate | Low | Low | Low | Moderate |
| Wu 2014 | Low | Low | Moderate | Low | Low | Low | Low |
| Wu 2016 | Low | Moderate | Low | Low | Moderate | Low | Moderate |
| Wu 2018 | Low | Moderate | Low | Low | Moderate | Low | Moderate |
| Xie 2020 | Low | Moderate | Low | Moderate | Low | Low | Moderate |
| Xing 2025* | Low | Low | Low | Moderate | Low | High | High |
| Yang 2024 | Low | Moderate | Low | Moderate | Moderate | Low | High |
| Yıldırım 2025* | Low | Low | Low | Moderate | Low | High | High |
| Zawavi 2024 | Low | Moderate | Moderate | Moderate | Moderate | Moderate | High |
| Zhang 2023* | Low | Moderate | Low | Moderate | Moderate | High | High |
| Zhang 2024* | Low | Moderate | Moderate | Moderate | Moderate | High | High |

*These studies reported incorrect effect sizes or 95% confidence intervals. Therefore, the data from these studies were not included in the final meta-analysis.

# Supplementary Table S6: GRADE of frequently evaluated prognostic factors

| **Summary of Findings table of pooled effect at follow up 1 year** | | | | | | | | | |
| --- | --- | --- | --- | --- | --- | --- | --- | --- | --- |
| **№ of studies** | **Certainty assessment** | | | | | | **Effect** | | **Certainty** |
|  | **Study design** | **Risk of bias** | **Inconsistency** | **Indirectness** | **Imprecision** | **Other considerations** | **Relative**  **(95% CI)** | **Absolute**  **(95% CI)** |  |
| Male | | | | | | | | | |
| 3 | NRSs | not serious | not serious | not serious | serious | none | **OR 0.61**  (0.29 to 1.31) | **1 fewer per 1,000**  (from 1 fewer to 0 fewer) | ⨁⨁⨁◯ Moderate |
| Nephrotic range proteinuria | | | | | | | | | |
| 2 | NRSs | serious | not serious | not serious | serious | none | **OR 3.38**  (1.60 to 7.16) | **3 fewer per 1,000**  (from 7 fewer to 2 fewer) | ⨁⨁◯◯ Low |

| **Summary of Findings table of pooled effect at follow up 1-2 years** | | | | | | | | | |
| --- | --- | --- | --- | --- | --- | --- | --- | --- | --- |
| **№ of studies** | **Certainty assessment** | | | | | | **Effect** | | **Certainty** |
|  | **Study design** | **Risk of bias** | **Inconsistency** | **Indirectness** | **Imprecision** | **Other considerations** | **Relative**  **(95% CI)** | **Absolute**  **(95% CI)** |  |
| Age | | | | | | | | | |
| 2 | NRSs | serious | not serious | not serious | not serious | none | **OR 1.24**  (1.04 to 1.47) | **1 fewer per 1,000**  (from 1 fewer to 1 fewer) | ⨁⨁⨁◯ Moderate |
| Anti-dsDNA | | | | | | | | | |
| 2 | NRSs | serious | not serious | not serious | serious | none | **OR 1.04**  (0.84 to 1.29) | **1 fewer per 1,000**  (from 1 fewer to 1 fewer) | ⨁⨁◯◯ Low |
| Biopsy class III | | | | | | | | | |
| 2 | NRSs | serious | very serious | not serious | very serious | none | **OR 1.11**  (0.24 to 5.14) | **1 fewer per 1,000**  (from 5 fewer to 0 fewer) | ⨁◯◯◯ Very low |
| Disease course | | | | | | | | | |
| 2 | NRSs | serious | not serious | not serious | serious | none | **OR 0.99**  (0.97 to 1.01) | **1 fewer per 1,000**  (from 1 fewer to 1 fewer) | ⨁⨁◯◯ Low |
| eGFR | | | | | | | | | |
| 2 | NRSs | serious | not serious | not serious | not serious | none | **OR 0.99**  (0.98 to 1.00) | **1 fewer per 1,000**  (from 1 fewer to 1 fewer) | ⨁⨁⨁◯ Moderate |
| Hemoglobin | | | | | | | | | |
| 2 | NRSs | serious | not serious | not serious | not serious | none | **OR 0.98**  (0.97 to 1.00) | **1 fewer per 1,000**  (from 1 fewer to 1 fewer) | ⨁⨁⨁◯ Moderate |

| **Summary of Findings table of pooled effect at follow up more than 2 years** | | | | | | | | | |
| --- | --- | --- | --- | --- | --- | --- | --- | --- | --- |
| **№ of studies** | **Certainty assessment** | | | | | | **Effect** | | **Certainty** |
|  | **Study design** | **Risk of bias** | **Inconsistency** | **Indirectness** | **Imprecision** | **Other considerations** | **Relative**  **(95% CI)** | **Absolute**  **(95% CI)** |  |
| Hypertension | | | | | | | | | |
| 2 | NRSs | serious | very serious | not serious | serious | none | **OR 7.61**  (1.76 to 32.88) | **8 fewer per 1,000**  (from 33 fewer to 2 fewer) | ⨁◯◯◯ Very low |
| Male | | | | | | | | | |
| 2 | NRSs | serious | very serious | not serious | very serious | none | **OR 2.49**  (0.22 to 28.15) | **2 fewer per 1,000**  (from 28 fewer to 0 fewer) | ⨁◯◯◯ Very low |
| Nephrotic range proteinuria | | | | | | | | | |
| 2 | NRSs | serious | not serious | not serious | serious | none | **OR 3.86**  (1.33 to 11.18) | **4 fewer per 1,000**  (from 11 fewer to 1 fewer) | ⨁⨁◯◯ Low |
| Scr | | | | | | | | | |
| 3 | NRSs | serious | serious | not serious | serious | none | **OR 1.92**  (0.83 to 4.46) | **2 fewer per 1,000**  (from 4 fewer to 1 fewer) | ⨁◯◯◯ Very low |
| Dialysis | | | | | | | | | |
| 2 | NRSs | not serious | not serious | not serious | very serious | none | **HR 12.18**  (2.33 to 63.68) | **12 fewer per 1,000**  (from 64 fewer to 2 fewer) | ⨁⨁◯◯ Low |
| eGFR <60ml/min/1.73m² | | | | | | | | | |
| 3 | NRSs | not serious | not serious | not serious | serious | none | **HR 2.86**  (1.18 to 6.98) | **3 fewer per 1,000**  (from 7 fewer to 1 fewer) | ⨁⨁⨁◯ Moderate |
| Hypertension | | | | | | | | | |
| 2 | NRSs | serious | serious | not serious | serious | none | **HR 0.82**  (0.24 to 2.74) | **1 fewer per 1,000**  (from 3 fewer to 0 fewer) | ⨁◯◯◯ Very low |
| Increased Scr | | | | | | | | | |
| 3 | NRSs | serious | not serious | not serious | serious | none | **HR 3.81**  (1.53 to 9.48) | **4 fewer per 1,000**  (from 9 fewer to 2 fewer) | ⨁⨁◯◯ Low |

**Continued**

| **Summary of Findings table of pooled effect at follow up more than 2 years** | | | | | | | | | |
| --- | --- | --- | --- | --- | --- | --- | --- | --- | --- |
| **№ of studies** | **Certainty assessment** | | | | | | **Effect** | | **Certainty** |
|  | **Study design** | **Risk of bias** | **Inconsistency** | **Indirectness** | **Imprecision** | **Other considerations** | **Relative**  **(95% CI)** | **Absolute**  **(95% CI)** |  |
| Kidney flare | | | | | | | | | |
| 3 | NRSs | not serious | serious | not serious | serious | none | **HR 3.18**  (1.07 to 9.48) | **3 fewer per 1,000**  (from 9 fewer to 1 fewer) | ⨁⨁◯◯ Low |
| Male | | | | | | | | | |
| 3 | NRSs | not serious | not serious | not serious | serious | none | **HR 2.96**  (1.75 to 5.00) | **3 fewer per 1,000**  (from 5 fewer to 2 fewer) | ⨁⨁⨁◯ Moderate |
| Renal non-remission at 6 months | | | | | | | | | |
| 3 | NRSs | not serious | not serious | not serious | serious | none | **HR 7.24**  (3.12 to 16.79) | **7 fewer per 1,000**  (from 17 fewer to 3 fewer) | ⨁⨁⨁◯ Moderate |
| Renal non-remission at 12 months | | | | | | | | | |
| 4 | NRSs | not serious | not serious | not serious | serious | none | **HR 3.92**  (1.98 to 7.78) | **4 fewer per 1,000**  (from 7 fewer to 2 fewer) | ⨁⨁⨁◯ Moderate |
| Treatment non-compliance | | | | | | | | | |
| 2 | NRSs | not serious | not serious | not serious | serious | none | **HR 1.92**  (0.93 to 3.94) | **2 fewer per 1,000**  (from 4 fewer to 1 fewer) | ⨁⨁⨁◯ Moderate |

# Supplementary Figure S1: Forest plot of the prevalence of renal non-remission

(a) Follow up within 1 year

(b) Follow up within 1-2 years

(c) Follow up over2 years

# Supplementary Figure S2: Forest plot of the prevalence of CKD

(a) Follow up within 1 year

(b) Follow up within 1-2 years

(c) Follow up over 2 years

# Supplementary Figure S3: Forest plot of the prevalence of ESRD

(a) Follow up within 1 year

(b) Follow up within 1-2 years

(c) Follow up within over 2 years

# Supplementary Figure S4: Forest plot of the prevalence of mortality

(a) Follow up within 1 year

(b) Follow up within 1-2 years

(c) Follow up within over 2 years

# Supplementary Figure S5: Forest plot of prognostic factors of poor renal outcomes within 1 year based on data from OR

(1) Age, years

(2) Male

(3) Non-White vs White

(4) Private insurance vs public insurance

(5) 24-hour urine protein, mg/kg/day

(6) Albumin, g/L

(7) Anti-dsDNA postive

(8) Blood urea nitrogen µmol/L

(9) CD4^+^/CD8^+^

(10) Disease course, months

(11) ECHC > 50%

(12) Edema

(13) eGFR, ml/min/1.73m^2^

(14) ESR, mm/h

(15) EULAR/ACR 2019 score <30, points

(16) IL-6, ng/L

(17) Nephrotic range proteinuria

(18) Number of extrarenal domain, ≥6

(19) SLEDAI score, points

(20) CYC vs CNIs

(21) CYC vs MMF

(22) EuroLupus regimen vs NIH regimen

(23) HCQ blood level, ng/ml

(24) MPA-AUC, mg/h/L

(25) MPA-AUC ≥45mg/h/L

(26) Use of ACEI

(27) Use of HCQ

# Supplementary Figure S6: Forest plot of prognostic factors of poor renal outcomes within 1-2 years based on data from OR

(1) Age, years

(2) Male

(3) 24-hour urine protein, mg/kg/day

(4) Active index, points

(5) Albumin, g/L

(6) Anti-dsDNA, IU/ml

(7) Biopsy class III vs other

(8) Biopsy class IV vs other

(9) Chronic index, points

(10) Complement 3, g/L

(11) Creatinine clearance rate, ml/min

(12) Disease course, months

(13) eGFR, ml/min/1.73m^2^

(14) Hemoglobin, g/L

(15) Hospital acquired infection

(16) Hypertension

(17) Invasive fungal infection

(18) Kidney flare

(19) NLR > 20

(20) Renal non-remission at 6 months

(21) Pulmonary infection

(22) Serum B-cell activating factor, pg/ml

(23) SLEDAI score, points

(24) Urinary protein/creatinine ratio, mg/mg

(25) Daily prednisolone dose at infection

# Supplementary Figure S7: Forest plot of prognostic factors of poor renal outcomes within 1-2 years based on data from HR

(1) Age, years

(2) eGFR, ml/min/1.73m^2^

(3) Hematological involvement

(4) Nephrotic range proteinuria

(5) Renal non-remission at 3 months

(6) Renal non-remission at 6 months

# Supplementary Figure S8: Forest plot of prognostic factors of poor renal outcomes over 2 years based on data from OR

(1) Age ≥ 10 years

(2) Age at dialysis initiation

(3) Male

(4) Active index, points

(5) Anti-dsDNA positive

(6) Biopsy class IV vs other

(7) Chronic index, points

(8) Clusterin/Urinary creatinine, per 1*10^-4^ increase

(9) Complement 4, g/L

(10) eGFR, ml/min/1.73m^2^

(11) eGFR <60ml/min/1.73m²

(12) Hematological involvement

(13) Hematuria 0~++

(14) Hypertension

(15) Hypoproteinemia

(16) IL-18, pg/ml

(17) Interstitial fibrosis

(18) Nephrotic range proteinuria

(19) Nervous-system involvement

(20) NGAL/Urinary creatinine, per 1*10^-4^ increase

(21) Scr, µmol/L

(22) SLEDAI score, points

(23) TFF3/Urinary creatinine, per 1*10^-4^ increase

(24) Time to acute dialysis discontinuation, days

(25) Tubular atrophy

(26) Treatment non-compliance

# Supplementary Figure S9: Forest plot of prognostic factors of poor renal outcomes over 2 years based on data from HR

(1) Age, years

(2) Age ≤ 13.1 years

(3) Age 9-12 vs ≤ 9 years

(4) Age 12-14 vs ≤ 9 years

(5) Age 14-18 vs ≤ 9 years

(6) Male

(7) 24-hour urine protein, mg/kg/day

(8) Acute renal failure

(9) Anti-phospholipid positivity

(10) Biopsy class II vs I

(11) Biopsy class III vs I

(12) Biopsy class IV vs I

(13) Biopsy class IV vs other

(14) Biopsy class V vs I

(15) Biopsy class V vs other

(16) CD19^+^ B cells, %

(17) CD3^+^ T cells, %

(18) Damage accrual

(19) Delayed SLE

(20) Dialysis

(21) eGFR < 60ml/min/1.73m²

(22) Glomerular sclerosis

(23) Hematological involvement

(24) Hypertension

(25) Hypoproteinemia

(26) IgG, g/L

(27) Increased Scr

(28) Kidney flare

(29) Nervous-system involvement

(30) NLR

(31) Renal non-remission at 6 months

(32) Renal non-remission at 12 months

(33) Number of renal flare, counts

(34) Pericarditis

(35) RPGN

(36) Serositis

(37) SLE diagnosis before 12

(38) SLEDAI score, points

(39) SLEDAI score >20

(40) Thrombotic microangiopathy

(41) Time to biopsy 16-30 days vs ≤ 15 days

(42) Time to biopsy1-3 months vs ≤ 15 days

(43) Time to biopsy >3 months vs ≤ 15 days

(44) AZA vs MMF

(45) MPA-AUC

(46) Others vs MMF

(47) Treatment non-compliance

(48) Treatment score, points

(49) BC, µg/m³

(50) NH4^+^, µg/m³

(51) NO3^-^, µg/m³

(52) OM, µg/m³

(53) PM2.5, µg/m³

(54) PM2.5 ≥ 30.09µg/m³

(55) SO4^2-^, µg/m³

References:

[1]	Asis C M L, Tee C A, Resontoc L P R. Prognostic factors for disease progression and mortality of childhood-onset lupus nephritis in the Philippines: a retrospective cohort study in a tertiary hospital. *Pediatr Rheumatol Online J*. (2025)23:86. *doi*: 10.1186/s12969-025-01128-w.

[2]	Andrade Balbi V, Artur Silva C, Nascimento Pedrosa T, Maria Rodrigues Pereira R, Maria de Arruda Campos L, Pires Leon E, et al. Hydroxychloroquine blood levels predicts flare in childhood-onset lupus nephritis. *Lupus*. (2022)31:97-104. *doi*: 10.1177/09612033211062515.

[3]	Chan E Y, Yap D Y, Wong W T, Wong W H, Wong S W, Lin K Y, et al. Long-Term Outcomes of Children and Adolescents With Biopsy-Proven Childhood-Onset Lupus Nephritis. *Kidney Int Rep*. (2023)8:141-50. *doi*: 10.1016/j.ekir.2022.10.014.

[4]	Chan E Y, Yap D Y, Wong W H, Wong S W, Lin K Y, Hui F Y, et al. Renal relapse in children and adolescents with childhood-onset lupus nephritis: a 20-year study. *Rheumatology (Oxford)*. (2024)63:953-61. *doi*: 10.1093/rheumatology/kead447.

[5]	Chen L, Zhang L, Liu B, Liu X, Huang Z, Tang K, et al. Exposure-response relationship of mycophenolic acid in pediatric lupus nephritis patients receiving multi-target therapy: An observational cohort study. *Semin Arthritis Rheum*. (2025)72:152674. *doi*: 10.1016/j.semarthrit.2025.152674.

[6]	Cheng C, Wen S, Lin Z, Jin B, Rong L, Chen L, et al. Treatment effectiveness and long‑term prognosis of childhood‑onset lupus nephritis. *Chin J Pediatr*. (2021)59:730-6.

[7]	Demir S, Gülhan B, Özen S, Çeleğen K, Batu E D, Taş N, et al. Long-term renal survival of paediatric patients with lupus nephritis. *Nephrol Dial Transplant*. (2022)37:1069-77. *doi*: 10.1093/ndt/gfab152.

[8]	Godron-Dubrasquet A, Woillard J B, Decramer S, Fila M, Guigonis V, Tellier S, et al. Mycophenolic acid area under the concentration-time curve is associated with therapeutic response in childhood-onset lupus nephritis. *Pediatr Nephrol*. (2021)36:341-7. *doi*: 10.1007/s00467-020-04733-x.

[9]	Y. G. Factors influencing the prognosis of children's lupus nephritis. *Chin J Pediatr*. (2001)39:18-20.

[10]	Han Y, Sha H, Zhou L, Yang Y, Wang Y, Yu Z, et al. A study of calcineurin inhibitors in comparison with intravenous cyclophosphamide for initial induction treatment of lupus nephritis class Ⅲ, Ⅳ, V in Chinese children. *Lupus*. (2025)34:961-71. *doi*: 10.1177/09612033251351338.

[11]	He G, Wang Y, Cheng C, Guo J, Lin Z, Liang Z, et al. PM(2.5) constituents associated with mortality and kidney failure in childhood-onset lupus nephritis: A 19-year cohort study. *Sci Total Environ*. (2024)949:175333. *doi*: 10.1016/j.scitotenv.2024.175333.

[12]	Jiao S, Zhao R, Chen Z, Shi Y, Ren D. Evaluation value of serum sTNF-R1, IGFBP-2, CFH on disease activity and prognosis of children with lupus nephritis. *Int J Lab Med*. (2022)43:945-9.

[13]	Lama M V, Deepthi B, Krishnasamy S, Ganesh R N, Srinivas B H, Rajappa M, et al. Clinical characteristics, disease flares, infections and their impact on kidney outcomes in pediatric lupus nephritis: A cohort study. *Pediatr Nephrol*. (2025)40:2529-38. *doi*: 10.1007/s00467-025-06666-9.

[14]	Li C, Han Y, Zhang L, Chen Z, Jin M, Sun S. Endocapillary hypercellularity levels are associated with early complete remission in children with class IV lupus nephritis as the initial presentation of SLE. *BMC Nephrol*. (2022)23:296. *doi*: 10.1186/s12882-022-02921-5.

[15]	Li Z, Wang J, Huang Y, Shen Q, Sun L, Xu H, et al. Identification of biomarkers that predict renal flare in childhood-onset lupus nephritis with mycophenolate acid. *Int Immunopharmacol*. (2023)117:109900. *doi*: 10.1016/j.intimp.2023.109900.

[16]	Liang S, Mei Z, Zeng Q. Prognosis Analysis of Childhood-onset Lupus Nephritis. *J Pract Med*. (2004)6:629-30.

[17]	Liu Y, Sun C. Correlation between of Levels of Serum HB-EGF and HIF-1α and its Prognosis in Children with Lupus Nephritis. *Journal of Clinical Research*. (2022)39:925-8. *doi*: 10.3969/j.issn.1671-7171.2022.06.033.

[18]	Liu H, Liu X, Zhao Y, Zhu F. Clinical and pathological features and prognosis of lupus nephritis complicated with thrombotic microangiopathy. *Chin J Appl Clin Pediatr*. (2024)39:203-8.

[19]	Lu X, Zhang Y. Relationship between peripheral blood lymphocyte subset characteristics and prognoses in children with lupus nephritis. *J Clin Nephrol*. (2024)24:494-501.

[20]	Luan L, Chen J. Analysis of Factors Affecting Kidney Survival Rate in Children with Lupus Nephritis. *Shandong Med J*. (2013)53:75-6.

[21]	Luan L, Wang Y. Diffuse proliferative lupus nephritis in children: Analysis on its prognostic risk factors. *Pract J Med Pharm*. (2013)30:778-81. *doi*: 10.14172/j.cnki.issn1671-4008.2013.09.049.

[22]	Luan L, Chen J, Li J. Impact of renal biopsy pathological parameters on survival rate of children with lupus nephritis, and their renal survival rate. *Pract J Med Pharm*. (2014)31:398-401. *doi*: 10.14172/j.cnki.issn1671-4008.2014.05.031.

[23]	De Mutiis C, Wenderfer S E, Basu B, Bagga A, Orjuela A, Sar T, et al. International cohort of 382 children with lupus nephritis - presentation, treatment and outcome at 24 months. *Pediatr Nephrol*. (2023)38:3699-709. *doi*: 10.1007/s00467-023-06018-5.

[24]	Park E, Jung J, Min J, Lee H, Park M J, Song J Y, et al. Long-term outcomes and associated prognostic risk factors of childhood-onset lupus nephritis. *Kidney Res Clin Pract*. (2025)44:389-95. *doi*: 10.23876/j.krcp.23.032.

[25]	Patrizi S T, Tandel M D, Boothroyd D, Simard J F, Hsu J J. Prognostic Value of the 2019 EULAR/American College of Rheumatology Systemic Lupus Erythematosus Classification Criteria to Renal Response One Year After Treatment in a Cohort With Childhood-Onset Lupus Nephritis. *ACR Open Rheumatol*. (2024)6:454-62. *doi*: 10.1002/acr2.11674.

[26]	Qiu S, Zhang H, Yu S, Yang Q, Zhang G, Yang H, et al. Clinical manifestations, prognosis, and treat-to-target assessment of pediatric lupus nephritis. *Pediatr Nephrol*. (2022)37:367-76. *doi*: 10.1007/s00467-021-05164-y.

[27]	Rianthavorn P, Prurapark P. Risk factors of infection-associated mortality in children with lupus nephritis in under-resourced areas. *Lupus*. (2019)28:1727-34. *doi*: 10.1177/0961203319882498.

[28]	Ruggiero B, Vivarelli M, Gianviti A, Benetti E, Peruzzi L, Barbano G, et al. Lupus nephritis in children and adolescents: results of the Italian Collaborative Study. *Nephrol Dial Transplant*. (2013)28:1487-96. *doi*: 10.1093/ndt/gfs589.

[29]	Smith E M D, Yin P, Jorgensen A L, Beresford M W. Clinical predictors of proteinuric remission following an LN flare - evidence from the UK JSLE cohort study. *Pediatr Rheumatol Online J*. (2018)16:14. *doi*: 10.1186/s12969-018-0230-4.

[30]	Smitherman E A, Chahine R A, Beukelman T, Lewandowski L B, Rahman A, Wenderfer S E, et al. Childhood-Onset Lupus Nephritis in the Childhood Arthritis and Rheumatology Research Alliance Registry: Short-Term Kidney Status and Variation in Care. *Arthritis Care Res (Hoboken)*. (2023)75:1553-62. *doi*: 10.1002/acr.25002.

[31]	Srivastava P, Abujam B, Misra R, Lawrence A, Agarwal V, Aggarwal A. Outcome of lupus nephritis in childhood onset SLE in North and Central India: single-centre experience over 25 years. *Lupus*. (2016)25:547-57. *doi*: 10.1177/0961203315619031.

[32]	Stotter B R, Cody E, Gu H, Daga A, Greenbaum L A, Duong M D, et al. Acute kidney injury requiring kidney replacement therapy in childhood lupus nephritis: a cohort study of the Pediatric Nephrology Research Consortium and Childhood Arthritis and Rheumatology Research Alliance. *Pediatr Nephrol*. (2023)38:1653-65. *doi*: 10.1007/s00467-022-05775-z.

[33]	Suhlrie A, Hennies I, Gellermann J, Büscher A, Hoyer P, Waldegger S, et al. Twelve-month outcome in juvenile proliferative lupus nephritis: results of the German registry study. *Pediatr Nephrol*. (2020)35:1235-46. *doi*: 10.1007/s00467-020-04501-x.

[34]	Taheri S, Beiraghdar F. Lupus nephritis in Iranian children: a review of 60 patients. *Ren Fail*. (2011)33:499-505. *doi*: 10.3109/0886022x.2011.573897.

[35]	Vachvanichsanong P, Dissaneewate P, McNeil E. Diffuse proliferative glomerulonephritis does not determine the worst outcome in childhood-onset lupus nephritis: a 23-year experience in a single centre. *Nephrol Dial Transplant*. (2009)24:2729-34. *doi*: 10.1093/ndt/gfp173.

[36]	Wang C S, Sadun R E, Zhou W, Miller K R, Pyle L, Ardoin S P, et al. Renal Response Outcomes of the EuroLupus and National Institutes of Health Cyclophosphamide Dosing Regimens in Childhood-Onset Proliferative Lupus Nephritis. *Arthritis Rheumatol*. (2024)76:469-78. *doi*: 10.1002/art.42725.

[37]	Suwannee Wisanuyotin M, Anirut Pattaragarn M, Ankanee Chanakul M, Adisorn Lumpaopong M. Outcomes of mycophenolate mofetil vs. intravenous cyclophosphamide in induction therapy of childhood-onset lupus nephritis. *J Med Assoc Thai*. (2020)103:785-90.

[38]	Wu J Y, Yeh K W, Huang J L. Early predictors of outcomes in pediatric lupus nephritis: focus on proliferative lesions. *Semin Arthritis Rheum*. (2014)43:513-20. *doi*: 10.1016/j.semarthrit.2013.07.005.

[39]	Wu C Y, Yang H Y, Yao T C, Liu S H, Huang J L. Serum IL-18 as biomarker in predicting long-term renal outcome among pediatric-onset systemic lupus erythematosus patients. *Medicine (Baltimore)*. (2016)95:e5037. *doi*: 10.1097/md.0000000000005037.

[40]	Wu C Y, Yang H Y, Chien H P, Tseng M H, Huang J L. Urinary clusterin-a novel urinary biomarker associated with pediatric lupus renal histopathologic features and renal survival. *Pediatr Nephrol*. (2018)33:1189-98. *doi*: 10.1007/s00467-018-3924-4.

[41]	Xie H, Zhu Q, Qin L, Ji C, Li L. The Evaluated Value of the Serum B-Cell Activating Factor to the Disease Activity and Prognosis in Children with Lupus Nephritis. *Labeled Immunoassays and Clinical Medicine*. (2020)27:1578-82+636.

[42]	Xing Y, Hu B, Zhang A. A Study on the Prediction of Poor Prognosis in Children with Lupus Nephritis by CD62P and CD4. *Medical Laboratory Science and Clinics*. (2025)36:59-64. *doi*: 10.3969/j.issn.1673-5013.2025.01.012.

[43]	Yang M, Liao Y, Jiang X, Cui J, Bi X, Zhao B. Changes in IL-6, sedimentation and CD4+/CD8+ levels in peripheral blood of children with lupus nephritis and their predictive value. *Medical Journal of West China*. (2024)36:433-7.

[44]	Gezgin Yıldırım D, Karaçayır N, Kısaoğlu H, Bülbül A Y, Garipçin P, Nalçacıoğlu H, et al. How have the clinical, laboratory, treatment features and outcomes in children with lupus nephritis progressed over the last 30 years? *Rheumatology (Oxford)*. (2025)64:4275-82. *doi*: 10.1093/rheumatology/keaf151.

[45]	Abdullah Zawavi M H, Nasir A, Van Rostenberghe H A, Ambak N J, Ilias M I. Predictive Factors and Renal Outcomes in Childhood Systemic Lupus Erythematosus. *Malaysian Journal of Medicine Health Sciences*. (2024)20:

[46]	Zhang P, Yang X, Fang X, Xu C, Gao C, Xia Z. Anti-C1q antibodies in lupus nephritis children with glomerular microthrombosis. *Nephrology (Carlton)*. (2023)28:485-94. *doi*: 10.1111/nep.14194.

[47]	Zhang P, Yang X, Fu M Z, Gao C L, Fang X, Xia Z K. Risk Factors and Clinical Outcomes of Renal Thrombotic Microangiopathy in Children with Lupus Nephritis in Terms of Pathological and Clinical Features. *Nephron*. (2024)148:609-17. *doi*: 10.1159/000538240.
